# Targeting monoamine oxidase A-regulated tumor-associated macrophage polarization for cancer immunotherapy

**DOI:** 10.1038/s41467-021-23164-2

**Published:** 2021-06-10

**Authors:** Yu-Chen Wang, Xi Wang, Jiaji Yu, Feiyang Ma, Zhe Li, Yang Zhou, Samuel Zeng, Xiaoya Ma, Yan-Ruide Li, Adam Neal, Jie Huang, Angela To, Nicole Clarke, Sanaz Memarzadeh, Matteo Pellegrini, Lili Yang

**Affiliations:** 1 Department of Microbiology, Immunology and Molecular Genetics, University of California, Los Angeles, CA USA; 2 Department of Molecular, Cell and Developmental Biology, and Institute for Genomics and Proteomics, University of California, Los Angeles, CA USA; 3 Eli and Edythe Broad Center of Regeneration Medicine and Stem Cell Research, University of California, Los Angeles, CA USA; 4 Department of Obstetrics and Gynecology, David Geffen School of Medicine, University of California Los Angeles, Los Angeles, CA USA; 5 The VA Greater Los Angeles Healthcare System, Los Angeles, CA USA; 6 Jonsson Comprehensive Cancer Center, the David Geffen School of Medicine, University of California, Los Angeles, CA USA; 7 Molecular Biology Institute, University of California, Los Angeles, CA USA

**Keywords:** Cancer immunotherapy, Tumour immunology

## Abstract

Targeting tumor-associated macrophages (TAMs) is a promising strategy to modify the immunosuppressive tumor microenvironment and improve cancer immunotherapy. Monoamine oxidase A (MAO-A) is an enzyme best known for its function in the brain; small molecule MAO inhibitors (MAOIs) are clinically used for treating neurological disorders. Here we observe MAO-A induction in mouse and human TAMs. MAO-A-deficient mice exhibit decreased TAM immunosuppressive functions corresponding with enhanced antitumor immunity. MAOI treatment induces TAM reprogramming and suppresses tumor growth in preclinical mouse syngeneic and human xenograft tumor models. Combining MAOI and anti-PD-1 treatments results in synergistic tumor suppression. Clinical data correlation studies associate high intratumoral *MAOA* expression with poor patient survival in a broad range of cancers. We further demonstrate that MAO-A promotes TAM immunosuppressive polarization via upregulating oxidative stress. Together, these data identify MAO-A as a critical regulator of TAMs and support repurposing MAOIs for TAM reprogramming to improve cancer immunotherapy.

## Abstract (web summary)

Monoamine oxidase A (MAO-A) is an outer mitochondrial membrane-bound enzyme best known for its function in the brain, but also linked to cancer progression. Here, the authors show that MAO-A is expressed in tumor associated macrophages, promoting their immunosuppressive properties, and that MAO inhibition suppresses tumor growth in preclinical models.

## Introduction

Over the past decade, cancer immunotherapy has achieved significant breakthroughs. In particular, immune checkpoint blockade (ICB) therapy has yielded remarkable clinical responses and revolutionised the treatment of many cancers^1^. So far, the FDA has approved cytotoxic T-lymphocyte antigen 4 (CTLA-4) and programmed cell death protein 1/ligand 1 (PD-1/PD-L1) blockade therapies for treating more than ten different malignancies^2^; however, only a small fraction of cancer patients respond to these therapies^3,4^. Most ICB therapies work through enhancing antitumor CD8^+^ T-cell responses, which can be greatly limited by the immunosuppressive tumor microenvironment (TME)^5^. Tumor-associated macrophages (TAMs), a key component of the immunosuppressive TME, dampen T-cell antitumor reactivity in the majority of solid tumors^6–9^. Growing evidence suggests that TAMs are responsible for inhibiting antitumor T-cell reactivity and limiting the ICB therapy efficacy, making TAMs potential targets for reversing the immunosuppressive TME and improving cancer immunotherapy^10–12^.

In general, TAMs are considered to mature from bone marrow-derived circulating monocytes; these monocytes are recruited to the tumor sites, exposed to chemokines and growth factors in the TME, and subsequently differentiate into TAMs^13,14^. There is also increasing evidence for tissue-resident macrophage-originated TAMs^15^. Depending on the surrounding immune environment, macrophages can be polarized towards an immunostimulatory phenotype by pro-inflammatory stimuli (e.g., IFN-γ) or towards an immunosuppressive phenotype by anti-inflammatory stimuli (e.g., IL-4 and IL-13)^16^. Although a binary polarization system is commonly used in macrophage studies, in most large-scale transcriptome analyses, TAMs showed a continuum of phenotypes expressing both immunostimulatory and immunosuppressive markers in addition to the extreme ends of polarization^16,17^. These mixed phenotypes and polarization states suggest the complexity of the TME and the residential TAM functionality. As a tumor develops, the enrichment of IL-4 and IL-13 produced by tumor cells and CD4^+^ T cells in the TME results in the polarization of TAMs towards an immunosuppressive phenotype, that promotes tumor growth, malignancy, and metastasis^16,18^. In established solid tumors, TAMs predominately exhibit an immunosuppressive phenotype, evidenced by their production of anti-inflammatory cytokines and arginase-1 (Arg1), as well as their expression of mannose receptor (CD206) and scavenger receptors^19,20^. Through metabolising l-arginine via Arg1, TAMs can directly suppress cytotoxic CD8^+^ T-cell responses^21,22^. Mannose receptor (CD206) expressed by TAMs can impair cytotoxicity of CD8^+^ T cells by suppressing CD45 phosphatase activity^23^. In addition, TAMs can inhibit T-cell activities through immune checkpoint engagement by expressing the ligands of the inhibitory receptors PD-1 and CTLA-4. For example, PD-L1 and PD-L2 expressed on TAMs interact with PD-1 of T cells to directly inhibit TCR signalling, cytotoxic function, and proliferation of CD8^+^ T cells^19^. These characteristics of TAMs make them potential targets for reversing the immunosuppressive TME to augment antitumor immunity.

Although the predominant phenotype of TAMs in established solid tumors is immunosuppressive, polarization is not fixed. Plasticity, one of the key features of TAMs, enables TAMs to change their phenotype in solid tumors and thereby providing a therapeutic window^24,25^. Repolarizing/reprogramming TAMs from an immunosuppressive and tumor-promoting phenotype towards an immunostimulatory and tumoricidal phenotype has thus become an attractive strategy in immunotherapy^18^. Preclinical and clinical studies are ongoing, evaluating TAM-repolarizing reagents (e.g., CD40 agonists, HDAC inhibitors, PI3Kγ inhibitors, creatine, etc.) for improving ICB therapy; certain efficacies have been reported^11,19,26^. Therefore, the search for new molecules regulating TAM polarization and the development of new combination treatments targeting TAM reprogramming is an active direction of current cancer immunotherapy studies.

Monoamine oxidase A (MAO-A) is an outer mitochondrial membrane-bound enzyme encoded by the X-linked *MAOA* gene. MAO-A is best known for its function in the brain, where it is involved in the degradation of a variety of monoamine neurotransmitters, including serotonin, dopamine, epinephrine, and norepinephrine. Through regulating the availability of serotonin, MAO-A modulates neuronal activities thereby influencing mood and behaviour in humans^27,28^. Through regulating the availability of dopamine and the abundance of dopamine breakdown by-product hydrogen peroxide (H_2_O_2_; hence oxidative stress), MAO-A is involved in multiple neurodegenerative diseases, including Parkinson’s disease (PD)^29,30^. FDA-approved small-molecule MAO inhibitors (MAOIs) are currently available for the treatment of neurological disorders, including depression and PD^30–32^. However, the functions of MAO-A outside of the brain are largely unknown.

In this study, we investigate the role of MAO-A in regulating TAM polarization and evaluate the possibility of repurposing MAOIs for reprogramming TAMs and improving cancer immunotherapy. We demonstrate that MAO-A promotes TAM immunosuppressive polarization and subsequent inhibition of antitumor immunity in mice via upregulating oxidative stress. MAOI treatment induces TAM reprogramming and suppresses tumor progression in preclinical mouse syngeneic and human xenograft tumor models. Combining MAOI and anti-PD-1 treatments result in synergistic tumor suppression. Clinical data correlation studies associate high intratumoral MAO-A expression with poor patient survival in a broad range of cancers. Together, these data identify MAO-A as a critical regulator of TAMs and support repurposing MAOIs for TAM reprogramming to improve cancer immunotherapy.

## Results

### MAO-A-deficient mice show reduced tumor growth associated with altered TAM polarization

**Fig. 1:**
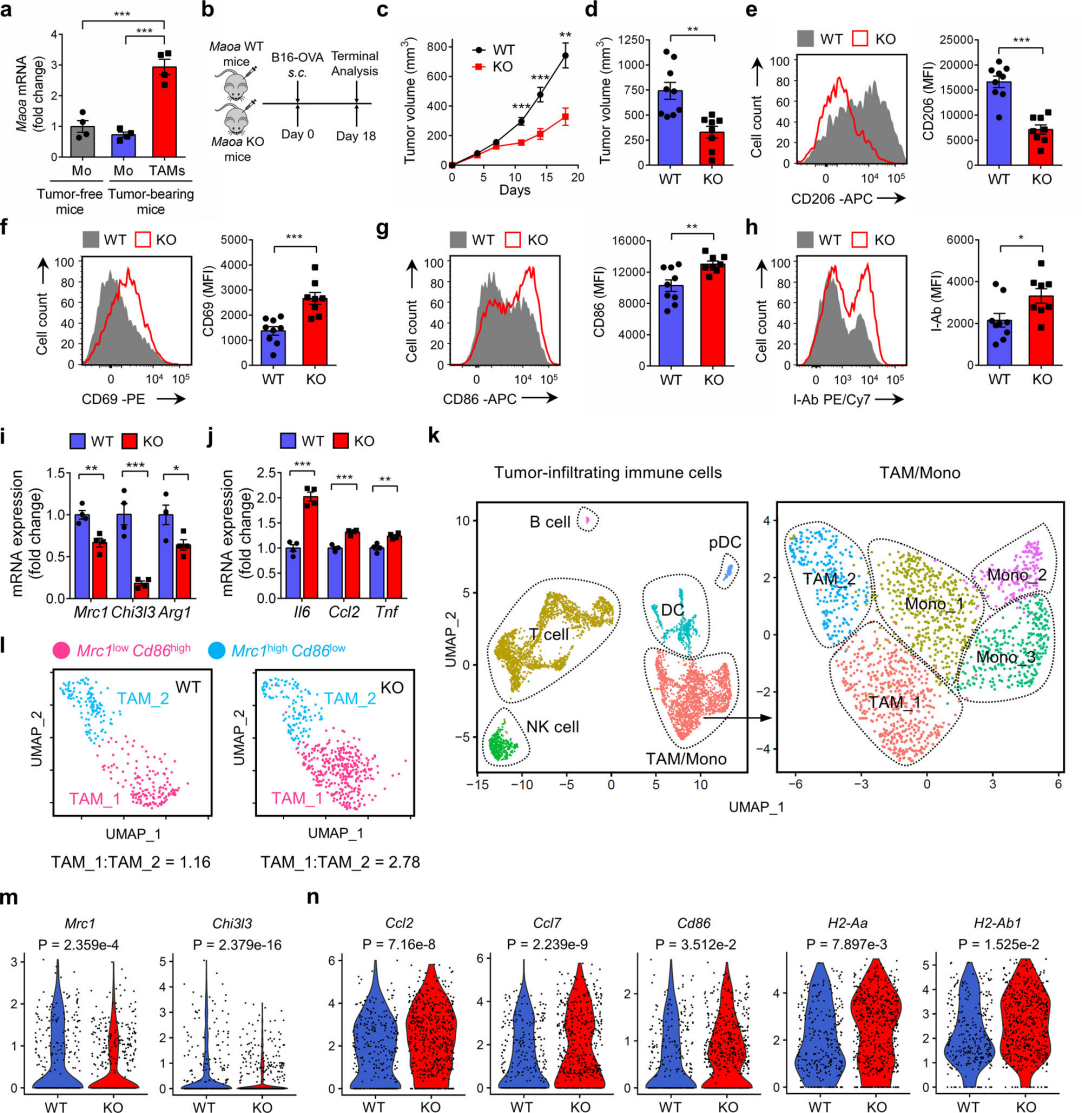
MAO-A-deficient mice show reduced tumor growth associated with altered TAM polarization. **a** QPCR analyses of *Maoa* mRNA expression in TAMs isolated from wild-type mice-bearing B16-OVA tumors. Monocytes (Mo) were isolated from peripheral blood of tumor-free and tumor-bearing mice (***p < 0.001). *N* = 4. **b**–**j** Studying B16-OVA tumor growth in *Maoa* WT and *Maoa* KO mice. **b** Experimental design. **c** Tumor growth (**p = 0.0038, ***p < 0.001). **d** Tumor volume at day 18 (**p = 0.0038). **e**–**h** FACS analyses of CD206 (**e**) (***p < 0.001), CD69 (**f**) (***p < 0.001), CD86 (**g**) (**p = 0.0064) and I-Ab (**h**) (*p = 0.0275) expression on TAMs at day 18. WT, *n* = 9; KO, *n* = 8. MFI mean fluorescence intensity. **i**, **j** QPCR analyses of immunosuppressive (*Mrc1*, **p = 0.0041; *Chi3l3*, ***p < 0.001 and *Arg1*, *p = 0.0339; **i**) and immunostimulatory (*Il6*, ***p < 0.001; *Ccl2*, ***p < 0.001 and *Tnf*, **p = 0.0036; **j**) signature genes mRNA expression in TAMs (*n* = 4). **k**–**n** scRNAseq analyses of tumor-infiltrating immune cells (TIIs) from *Maoa* WT and *Maoa* KO mice at day 14 post B16-OVA tumors challenge. **k** Uniform Manifold Approximation and Projection (UMAP) of single TIIs showing the formation of six cell clusters (TAM/Mono, T cell, NK cell, B cell, DC and pDC) from total CD45.2^+^ TIIs and five cell clusters (TAM_1, TAM_2, Mono_1, Mono_2 and Mono_3) from the TAM/Mono subpopulation. Each dot represents one single cell and is coloured according to cell types. Mono monocyte, NK natural killer cell, DC dendritic cell, pDC plasmacytoid dendritic cell. **l** UMAP of the TAM subpopulation, showing the formation of two clusters (TAM_1: *Mrc1*^low^*Cd86*^high^; and TAM_2: *Mrc1*^high^*Cd86*^low^). Each dot represents one single cell and is coloured according to cell clusters. Ratios of TAM_1:TAM_2 are presented. **m**, **n** Violin plots of immunosuppressive (*Mrc1* and *Chi3l3*; **m**) and immunostimulatory (*Ccl2*, *Ccl7*, *Cd86*, *H2-Aa*, and *H2-Ab1*; **n**) signature genes expression in single TAMs. Each dot represents an individual cell. Representative of 1 (**k**–**n**), 3 (**a**), and 5 (**b**–**j**) experiments. Analysed by one-way ANOVA (**a**) or by Student’s *t* test (**c**–**j**). *p* values of violin plots are determined by Wilcoxon rank-sum test (**m**, **n**). Statistics are all two-sided. Source data are provided as a Source Data file.

In a search for new molecules regulating TAM reprogramming, we inoculated C57BL/6J mice with syngeneic B16-OVA melanoma tumors, isolated TAMs and assessed TAM gene expression profiles. Monocytes isolated from tumor-free and tumor-bearing mice were included as controls. In addition to changes in classical genes involved in regulating macrophage immune responses, we observed the induction of a *Maoa* gene in TAMs (Fig. 1a), suggesting that MAO-A may be involved in modulating TAM activities.

To study the role of MAO-A in antitumor immunity in vivo, we used MAO-A-deficient mice that carry a hypomorphic MAO-A mutant^33^. Although a degree of *Maoa* expression leakage in the brain had been previously reported in these mice^33^, analysis of their immune system showed nearly complete ablation of MAO-A expression in major lymphoid organs, including the spleen and bone marrow (BM) (Supplementary Fig. 1a). Since we focused on immune cells in this study, we denote these mice as *Maoa* knockout (KO) mice. When challenged with B16-OVA melanoma cells (Fig. 1b), tumor growth in *Maoa* KO mice was significantly suppressed compared to that in *Maoa* wild-type (WT) mice (Fig. 1c, d). Although similar levels of TAMs (gated as CD45.2^+^CD11b^+^Ly6G^−^Ly6C^−/low^F4/80^+^ cells) were detected in *Maoa* WT and *Maoa* KO mice (Supplementary Fig. 1b, c), compared to their WT counterparts, TAMs isolated from *Maoa* KO mice exhibited a less immunosuppressive phenotype, indicated by their decreased expression of immunosuppressive markers (i.e., CD206; Fig. 1e), and their increased expression of immunostimulatory molecules (i.e., CD69, CD86 and MHC class II I-Ab; Fig. 1f–h). Further analysis showed that TAMs from *Maoa* KO mice expressed reduced levels of immunosuppression-associated genes (i.e., *Mrc1*, *Chi3l3* and *Arg1*; Fig. 1i) and increased levels of pro-inflammatory cytokine genes (i.e., *Il6*, *Tnfα* and *Ccl2*; Fig. 1j). Corresponding to the altered TAM polarization in *Maoa* KO mice, tumor-infiltrating CD8^+^ T cells in these mice showed enhanced activation (i.e., increased production of Granzyme B; Supplementary Fig. 1d). Single-cell RNA sequencing (scRNAseq) analysis was performed on tumor-infiltrating immune cells (TIIs) isolated from *Maoa* WT and *Maoa* KO mice. Uniform Manifold Approximation and Projection (UMAP) of total TIIs showed the formation of 6 cell clusters (TAM/Mono, T cell, NK cell, B cell, DC and pDC; Fig. 1k and Supplementary Fig. 1e); cell cluster distributions were similar comparing *Maoa* WT and KO TIIs (Supplementary Fig. 1f, g). Further UMAP analysis of the TAM/Mono subpopulation showed the formation of five cell clusters (TAM_1, TAM_2, Mono_1, Mono_2 and Mono_3; Fig. 1k and Supplementary Fig. 1h). Compared to that in *Maoa* WT mice, the TAM subpopulation in *Maoa* KO mice comprised an increased ratio of TAM_1 (*Mrc1*^low^*Cd86*^high^) to TAM_2 (*Mrc1*^high^*Cd86*^low^) cells, corresponding to a reduced immunosuppressive phenotype of TAMs in *Maoa* KO mice (Fig. 1l and Supplementary Fig. 1i); the monocyte subpopulation in *Maoa* WT and KO mice comprised a similar composition of Mono_1 (*Ly6c2*^med^), Mono_2 (*Ly6c2*^lo^), and Mono_3 (*Ly6c2*^hi^) cells (Supplementary Fig. 1j, k). Gene expression profile analysis confirmed a reduction of TAMs expressing immunosuppressive genes (i.e., *Mrc1* and *Chi3I3*; Fig. 1m) and an enrichment of TAMs expressing immunostimulatory genes (i.e., *Ccl2*, *Ccl7*, *Cd86*, *H2-Aa*, and *H2-Ab1*; Fig. 1n) in *Maoa* KO mice. These data strongly indicate that MAO-A is involved in regulating TAM polarization thereby modulating antitumor immunity.

### MAO-A directly regulates TAM polarization and influences TAM-associated T-cell antitumor reactivity

**Fig. 2:**
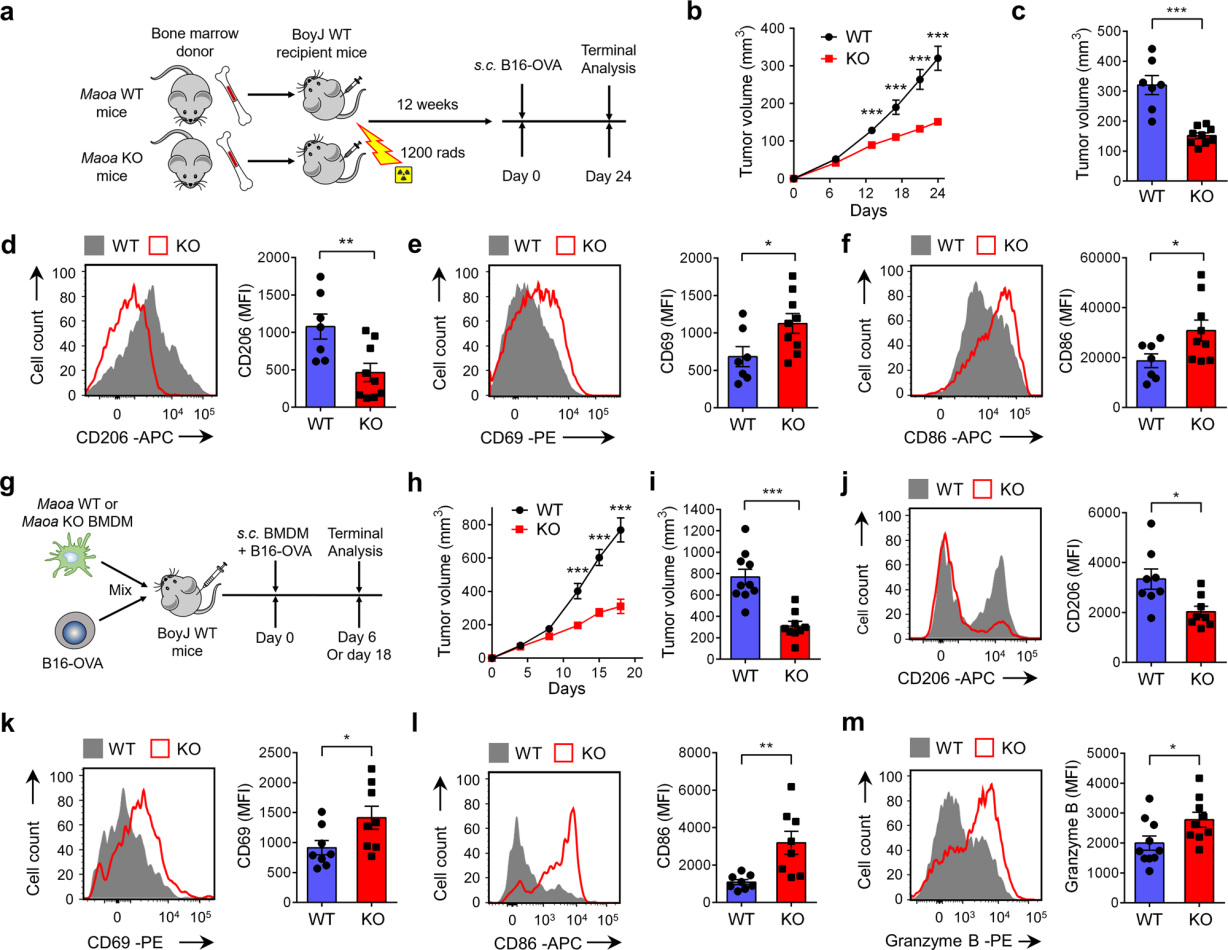
MAO-A directly regulates TAM polarization and influences TAM-associated antitumor T-cell reactivity. **a**–**f** Studying B16-OVA tumor growth and TAM phenotype in BoyJ (CD45.1) wild-type mice reconstituted with bone marrow cells isolated from either *Maoa* WT or *Maoa* KO donor mice (denoted as WT or KO experimental mice, respectively). **a** Experimental design. **b** Tumor growth (***p < 0.001). **c** Tumor volume at day 24 (***p < 0.001). **d**–**f** FACS analyses of CD206 (**d**) (**p = 0.0087), CD69 (**e**) (*p = 0.0349) and CD86 (*p = 0.0429). **f** Expression on TAMs at day 24. WT, *n* = 7; KO, *n* = 9. **g**–**m** Studying B16-OVA tumor growth and antitumor T-cell reactivity in a tumor-TAM co-inoculation in vivo experiment. BoyJ wild-type mice received s.c. inoculation of B16-OVA tumor cells mixed with either *Maoa* WT or *Maoa* KO BMDMs (denoted as WT or KO experimental mice, respectively). BMDM bone marrow-derived macrophage. **g** Experimental design. **h** Tumor growth (*n* = 9–10) (***p < 0.001). **i** Tumor volume at day 18 (***p < 0.001). WT, *n* = 10; KO, *n* = 9. **j**, **l** FACS analyses of CD206 (**j**) (*p = 0.0139), CD69 (**k**) (*p = 0.0443) and CD86 (**l**) (**p = 0.0047) expression on CD45.2^+^ TAMs at day 6 (*n* = 8). **m** FACS analyses of intracellular Granzyme B production in tumor-infiltrating CD45.1^+^CD8^+^ T cells at day 18 (*p = 0.0371) (WT, *n* = 10; KO, *n* = 9). Representative of three experiments. Analysed by Student’s *t* test. Statistics are all two-sided. Source data are provided as a Source Data file.

In our *Maoa* KO mice tumor challenge study, MAO-A deficiency impacted both immune and non-immune cells (Fig. 1b). To determine whether MAO-A directly regulates immune cells, we conducted a BM transfer experiment wherein BM cells harvested from *Maoa* WT or KO mice were adoptively transferred into BoyJ (CD45.1) WT-recipient mice followed by B16-OVA tumor challenge (Fig. 2a). In this experiment, MAO-A deficiency comparison was confined to immune cells. MAO-A deficiency in immune cells resulted in suppressed tumor growth (Fig. 2b, c), altered TAM polarization (i.e., downregulation of immunosuppressive markers such as CD206, Fig. 2d; and upregulation of immunostimulatory markers such as CD69, CD86 and MHC class II I-Ab; Fig. 2e, f and Supplementary Fig. 2a), and enhanced tumor-infiltrating CD8^+^ T-cell activation (i.e., increased production of cytotoxic molecules such as Granzyme B; Supplementary Fig. 2b), indicating that MAO-A directly regulates immune cell antitumor activity, in particular TAM polarization and T-cell antitumor reactivity.

To further study whether MAO-A acts as a macrophage autonomous factor directly regulating TAM polarization and thereby influencing antitumor immunity, we performed a macrophage adoptive transfer tumor experiment. BM cells were harvested from *Maoa* WT and KO mice then cultured into bone marrow-derived macrophages (BMDMs). These *Maoa* WT or KO BMDMs were then mixed with B16-OVA melanoma cells and subcutaneously (s.c.) injected into BoyJ WT-recipient mice to establish solid tumors (Fig. 2g). In this study, MAO-A-deficiency comparison was confined to TAMs. Suppressed tumor growth (Fig. 2h, i), downregulated expression of TAM immunosuppressive markers (i.e., CD206; Fig. 2j), upregulated expression of TAM immunostimulatory markers (i.e., CD69 and CD86; Fig. 2k, l) and enhanced tumor-infiltrating CD8^+^ T-cell reactivity (i.e., increased production of Granzyme B; Fig. 2m) were observed in mice receiving *Maoa* KO BMDMs. Collectively, these in vivo studies demonstrate that MAO-A acts as an autonomous factor directly regulating TAM polarization, and thereby influencing T-cell antitumor reactivity and impacting tumor growth.

### MAO-A promotes macrophage immunosuppressive polarization

**Fig. 3:**
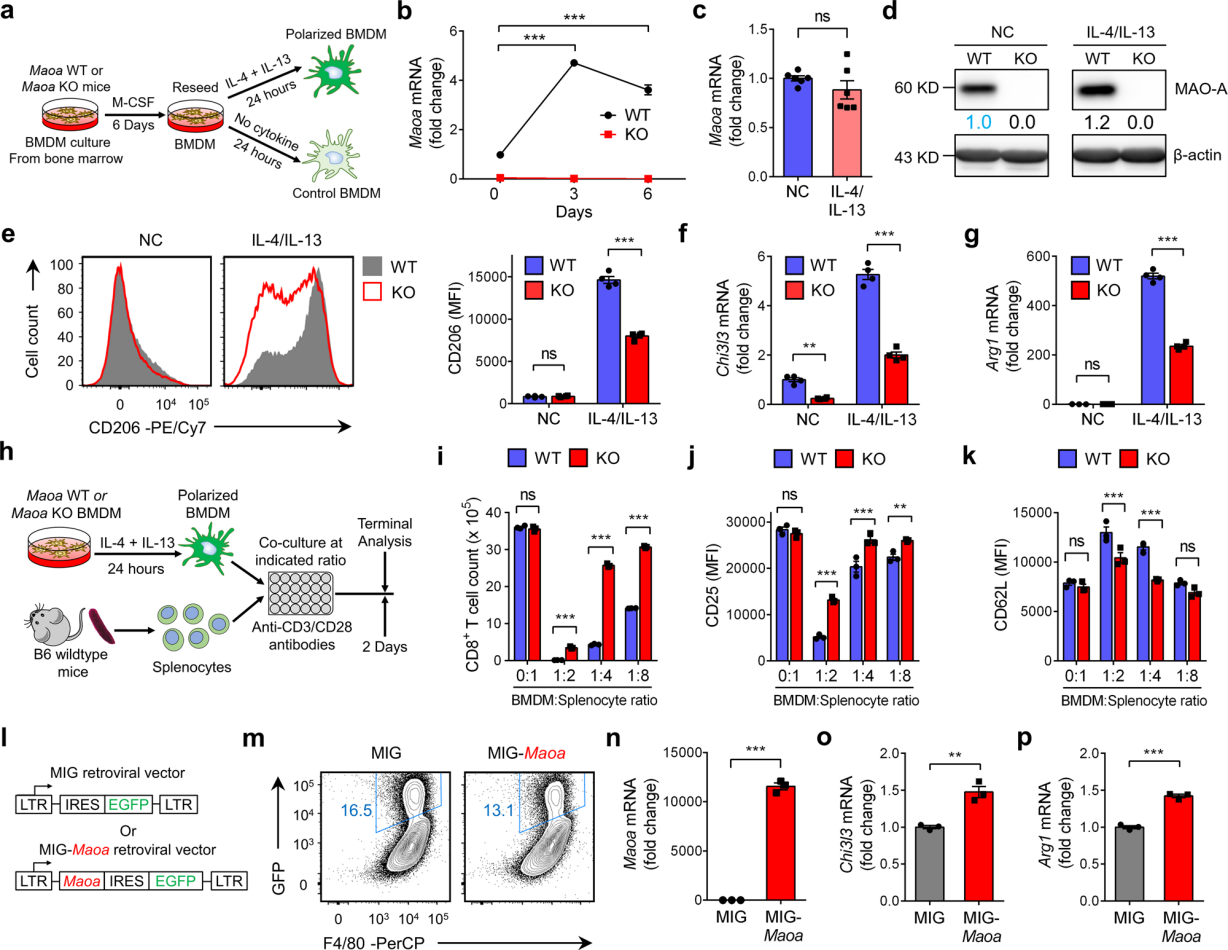
MAO-A promotes macrophage immunosuppressive polarization. **a**–**g** Studying the in vitro differentiation and IL-4/IL-13-induced polarization of *Maoa* WT (WT) and *Maoa* KO (KO) BMDMs. **a** Experimental design. **b**, **c** QPCR analyses of *Maoa* mRNA expression over the 6-day BMDM differentiation culture (**b**) (***p < 0.001) and IL-4/IL-13-induced polarization (**c**) (*n* = 6). **d** Western blot analyses of MAO-A protein expression in the indicated BMDMs. Source data are provided as a Source Data file. **e** FACS analyses of CD206 expression on the indicated BMDMs (***p < 0.001) (*n* = 4). **f**, **g** QPCR analyses of *Chi3l3* (**f**) (NC, **p = 0.0023; IL-4/IL-13, ***p < 0.001) and *Arg1* (**g**) (***p < 0.001) mRNA expression in the indicated BMDMs (*n* = 4). NC no cytokine control BMDMs, IL-4/IL-13 IL-4 and IL-13-polarized BMDMs, ns not significant. **h**–**k** Studying the T-cell suppression function of *Maoa* WT (WT) and *Maoa* KO (KO) IL-4/IL-13-polarized BMDMs in an in vitro macrophage/T-cell co-culture assay (*n* = 3). **h** Experimental design. **i** FACS quantification of CD8^+^ T cells (identified as TCRβ^+^CD4^-^CD8^+^ cells) (***p < 0.001). **j**, **k** FACS analyses of CD25 (**j**) (1:2, ***p < 0.001; 1:4, ***p < 0.001; 1:8, **p = 0.0038) and CD62L (**k**) (***p < 0.001) expression on CD8^+^ T cells. **l**–**p** Studying the IL-4/IL-13-induced polarization of *Maoa* KO BMDMs with MAO-A overexpression (*n* = 3). In vitro-cultured *Maoa* KO BMDMs were transduced with either a MIG-*Maoa* retrovector or a MIG mock retrovector, polarized with IL-4/IL-13, followed by FACS sorting of GFP^+^ *Maoa* KO BMDMs for further analyses. **l** Schematics of the MIG and MIG-*Maoa* retrovectors. **m** FACS analyses of prior-to-sorting *Maoa* KO BMDMS, showing retrovector transduction efficiency (measured as %GFP^+^ cells). **n**–**p** QPCR analyses of sorted GFP^+^ *Maoa* KO BMDMs, showing the mRNA expression of *Maoa* (**n**) (***p < 0.001), *Chi3l3* (**o**) (**p = 0.0038), and *Arg1* (**p**) (***p < 0.001). Representative of three (**h**–**k**, **l**–**p**) and four (**a**–**g**) experiments. ns not significant. Analysed by one-way ANOVA (**b**), two-way ANOVA (**e**–**g, i**–**k**) or by Student’s *t* test (**c**, **n**–**p**). Statistics are all two-sided. Source data are provided as a Source Data file.

To study MAO-A regulation of macrophage polarization, we cultured *Maoa* WT and KO BMDMs in vitro and polarized these macrophages towards an immunosuppressive phenotype by adding anti-inflammatory stimuli (i.e., IL-4 and IL-13; Fig. 3a). We observed a sharp induction of *Maoa* mRNA expression in *Maoa* WT BMDMs during M-CSF-induced macrophage differentiation; *Maoa* expression was then plateaued in matured BMDMs and maintained over IL-4/IL-13-induced immunosuppressive polarization (Fig. 3b, c). MAO-A expression was undetectable in *Maoa* KO BMDMs, confirming their *Maoa*-deficiency genotype (Fig. 3b, d). Compared to their wild-type counterpart, *Maoa* KO macrophages displayed a less immunosuppressive phenotype under IL-4/IL-13 stimulation, evidenced in their reduced expression of immunosuppressive markers (i.e., CD206; Fig. 3e) and signature genes (i.e., *Chi3l3* and *Arg1*; Fig. 3f, g and Supplementary Fig. 3a). When tested in a macrophage/T-cell co-culture assay (Fig. 3h), in agreement with their less immunosuppressive phenotype, IL-4/IL-13-polarized *Maoa* KO macrophages exhibited impaired suppression of wild-type CD8^+^ T cells under anti-CD3/CD28 stimulation, shown as their attenuated inhibition of CD8^+^ T-cell proliferation (Fig. 3i) and activation marker expression (i.e., upregulation of CD25 and CD44, and downregulation of CD62L; Fig. 3j, k and Supplementary Fig. 3b).

To verify whether MAO-A deficiency directly contributed to the alleviated immunosuppressive polarization of *Maoa* KO macrophages, we performed a rescue experiment. We constructed a MIG-*Maoa* retroviral vector, used this vector to transduce *Maoa* KO BMDMs, and achieved overexpression of MAO-A in these macrophages (Fig. 3l–n and Supplementary Fig. 3c). MAO-A overexpression significantly exacerbated the immunosuppressive phenotype of IL-4/IL-13-stimulated *Maoa* KO BMDMs (i.e., upregulation of immunosuppressive signature genes such as *Chi3l3* and *Arg1*; Fig. 3o, p). Taken together, these results indicate that MAO-A acts as an autonomous factor promoting macrophage immunosuppressive polarization under anti-inflammatory stimuli.

### MAO-A promotes macrophage immunosuppressive polarization via ROS upregulation

**Fig. 4:**
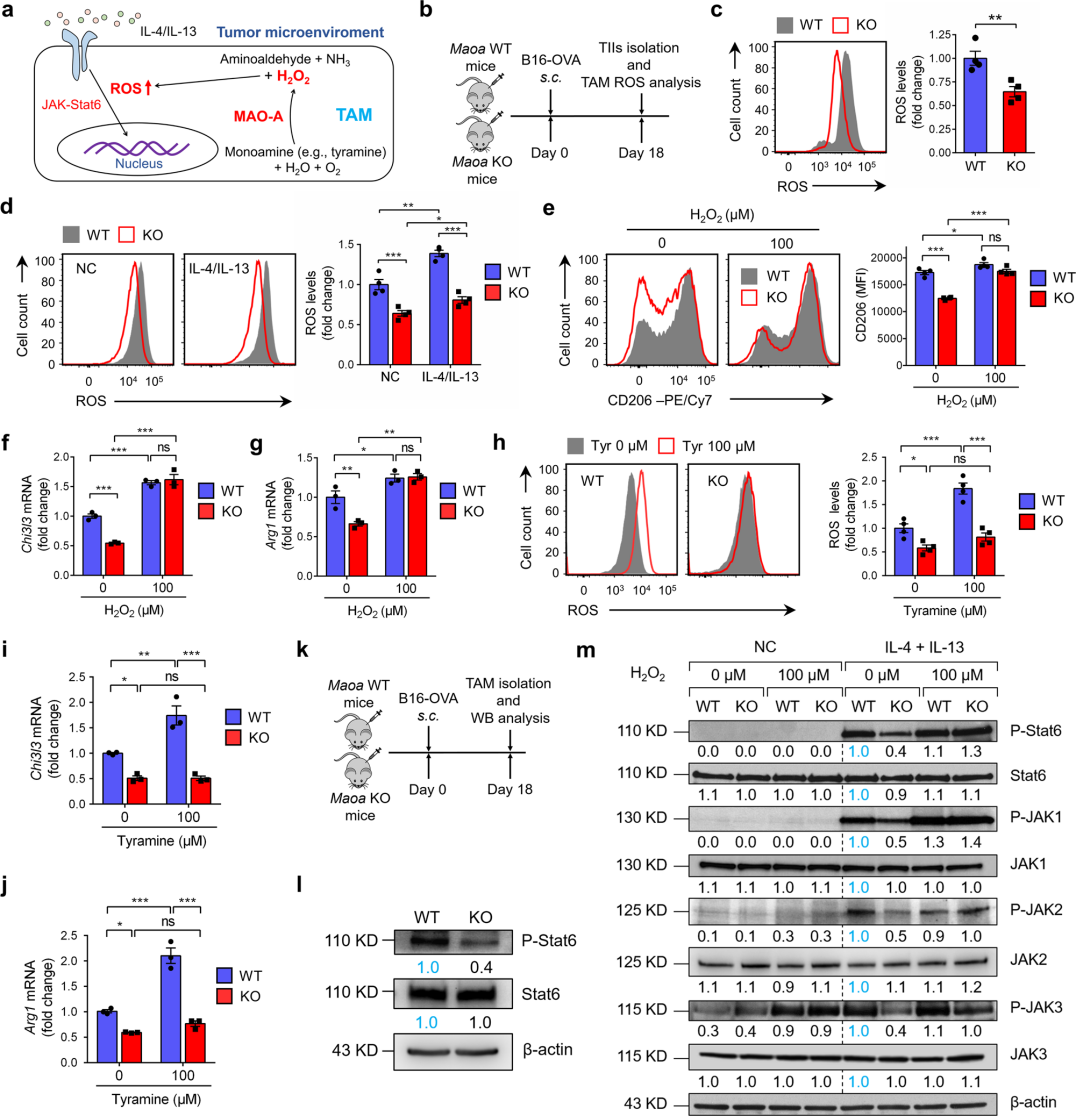
MAO-A promotes macrophage immunosuppressive polarization via ROS upregulation. **a** Schematics showing MAO-A breaks down monoamines and generates hydrogen peroxide (H_2_O_2_) as a by-product, thereby increasing reactive oxygen species (ROS) levels in a TAM. **b**, **c** Studying the in vivo ROS levels in TAMs isolated from *Maoa* WT and *Maoa* KO mice-bearing B16-OVA tumors (*n* = 4). **b** Experimental design. **c** FACS analyses of ROS levels in TAMs at day 18. TAMs were gated as the CD45.2^+^CD11b^+^Ly6G^−^Ly6C^-/low^F4/80^+^ cells of total TIIs (**p = 0.0088). **d** FACS analyses of ROS levels in in vitro-cultured *Maoa* WT and *Maoa* KO BMDMs, without or without IL-4/IL-13 polarization (*n* = 4). NC no cytokine, IL-4/IL-13 IL-4/IL-13-polarized. *p < 0.05, **p < 0.01 and ***p < 0.001. **e**–**g** Study of IL-4/IL-13-polarized *Maoa* WT and *Maoa* KO BMDMs, with or without H_2_O_2_ treatment (*n* = 3). **e** FACS analyses of CD206 expression. **f**, **g** QPCR analyses of *Chi3l3* (**f**) and *Arg1* (**g**) mRNA expression. *p < 0.05, **p < 0.01 and ***p < 0.001. **h**–**j** Study of IL-4/IL-13-polarized *Maoa* WT and *Maoa* KO BMDMs, with or without tyramine supplement (*n* = 3). **h** FACS analyses of ROS levels. **i**, **j** QPCR analyses of *Chi3l3* (**i**) and *Arg1* (**j**) mRNA expression. *p < 0.05, **p < 0.01 and ***p < 0.001. **k**, **l** Study of TAMs isolated from *Maoa* WT and *Maoa* KO mice-bearing B16-OVA tumors at day 18 (combined from five mice per group). **k** Experimental design. **l** Western blot analyses of TAMs. TAMs were FACS sorted as the DAPI^−^CD45.2^+^CD11b^+^Ly6G^−^Ly6C^−/low^F4/80^+^ cells from total TIIs. Source data are provided as a Source Data file. **m** Western blot analyses of JAK-Stat6 signalling in *Maoa* WT and *Maoa* KO BMDMs, with or without IL-4/IL-13 polarization and H_2_O_2_ treatment. BMDMs were treated with H_2_O_2_ for 30 min prior to IL-4/IL-13 stimulation for another 30 min. Representative of three experiments. Analysed by two-way ANOVA (**d**–**j**) or by Student’s *t* test (**c**). Statistics are all two-sided. Source data are provided as a Source Data file.

Next, we sought to investigate the molecular mechanisms regulating MAO-A promotion of macrophage immunosuppressive polarization. It has been reported that intracellular reactive oxygen species (ROS; hence, oxidative stress) elicit macrophage immunosuppressive features^34–36^. MAO-A catalyzes the oxidative deamination of monoamines, thereby generating hydrogen peroxide (H_2_O_2_) as a by-product that can increase intracellular ROS levels. We, therefore, speculated that MAO-A might promote TAM immunosuppressive polarization in TME via upregulating ROS levels in TAMs (Fig. 4a).

To test this hypothesis, we directly measured ROS levels in TAMs isolated from *Maoa* WT and KO mice-bearing B16-OVA tumors and detected significantly lower levels of ROS in *Maoa* KO TAMs (Fig. 4b, c). Measurement of ROS levels in in vitro-cultured *Maoa* WT and KO BMDMs also showed reduced levels of ROS in *Maoa* KO BMDMs, with or without IL-4/IL-13 stimulation, in agreement with the in vivo TAM results (Fig. 4d). Supplementing H_2_O_2_ to IL-4/IL-13-stimulated *Maoa* WT and KO BMDMs elevated their intracellular ROS to similar levels (Supplementary Fig. 4a, b) and eliminated their differences in expression of immunosuppressive markers (i.e., CD206; Fig. 4e) and signature genes (i.e., *Chi3l3* and *Arg1*; Fig. 4f, g).

On the other hand, supplementation of tyramine, a substrate of MAO-A, increased ROS levels and upregulated the expression of immunosuppressive genes (i.e., *Chi3l3* and *Arg1*) in *Maoa* WT BMDMs but not in *Maoa* KO BMDMs (Fig. 4h–j). Taken together, these data indicate that MAO-A regulates macrophage immunosuppressive polarization via modulating macrophage intracellular ROS levels.

The JAK-Stat6 signalling pathway plays a key role in mediating IL-4/IL-13-induced immunosuppressive polarization of TAMs in TME^37,38^. After IL-4/IL-13 stimulation, JAK is phosphorylated and subsequently phosphorylates Stat6; phosphorylated Stat6 dimerises and migrates to the nucleus, where it binds to the promoters of IL-4 and IL-13 responsive genes including those involved in macrophage immunosuppressive functions^39^. ROS has been reported to promote JAK and Stat6 phosphorylation in a variety of cell types^40,41^. Since we observed decreased ROS levels in *Maoa* KO macrophages compared to those in *Maoa* WT macrophages (Fig. 4b, c), we postulated that MAO-A may impact macrophage polarization through upregulating ROS levels and thereby sensitising the JAK-Stat6 signalling pathway. Indeed, direct analysis of TAMs isolated from B16-OVA tumor-bearing *Maoa* WT and *Maoa* KO mice confirmed that compared to wild-type TAMs, MAO-A-deficient TAMs showed reduced Stat6 activation (i.e., reduced Stat6 phosphorylation; Fig. 4k, l). Further analysis of IL-4/IL-13-induced JAK-Stat6 signalling pathway in *Maoa* KO BMDMs compared to that in *Maoa* WT BMDMs showed significantly reduced JAK-Stat6 signalling (i.e., reduced JAK1, JAK2, JAK3 and Stat6 phosphorylation; Fig. 4m). Supplementing H_2_O_2_ to IL-4/IL-13-stimulated *Maoa* WT and KO BMDMs increased their JAK-Stat6 signalling to similar levels (i.e., comparable JAK1, JAK2, JAK3 and Stat6 phosphorylation; Fig. 4m), corresponding to their comparable high levels of ROS (Supplementary Fig. 4a, b). These data indicate that MAO-A promotes macrophage immunosuppressive polarization via ROS-sensitised JAK-Stat6 pathway activation.

Collectively, these in vivo and in vitro data support a working model that MAO-A promotes TAM immunosuppressive polarization in TME, at least partly through upregulating TAM intracellular ROS levels and thereby enhancing the IL-4/IL-13-induced JAK-Stat6 signalling pathway.

### MAO-A blockade for cancer immunotherapy—syngeneic mouse tumor model studies

**Fig. 5:**
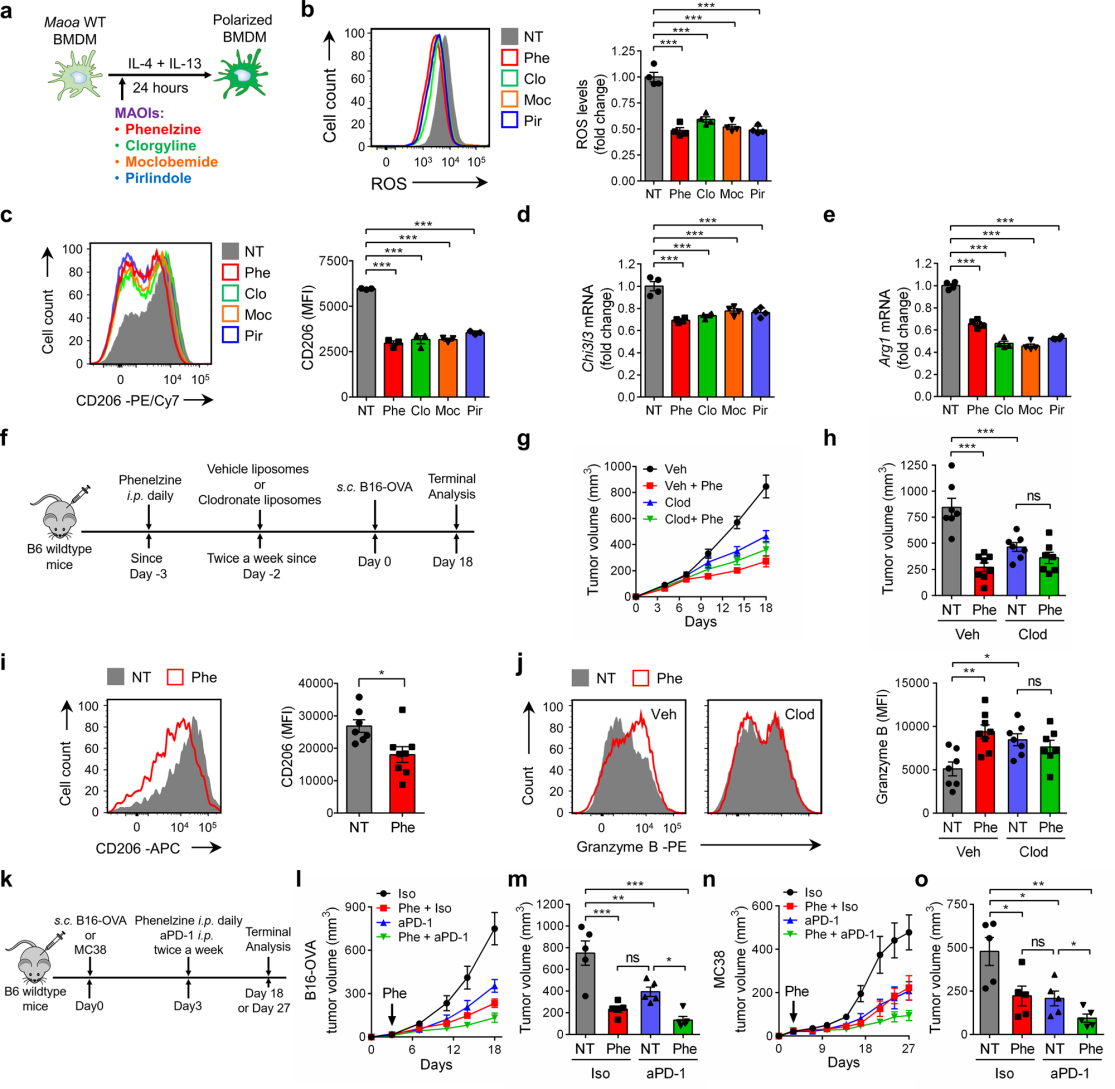
MAO-A blockade for cancer immunotherapy—syngeneic mouse tumor model studies. **a**–**e** Studying the effect of MAOI treatment on IL-4/IL-13-induced BMDM polarization in vitro (*n* = 4). **a** Experimental design. Wild-type BMDMs were stimulated with IL-4/IL-13 with or without MAOI treatment. MAOIs (monoamine oxidase inhibitors) studied were phenelzine (Phe; 20 μM), clorgyline (Clo; 20 μM), moclobemide (Moc; 200 μM), and pirlindole (Pir; 20 μM). NT no MAOI treatment. **b** FACS analyses of ROS levels in BMDMs. **c** FACS analyses of CD206 expression on BMDMs. **d**, **e** QPCR analyses of *Chi3l3* (**d**) and *Arg1* (**e**) mRNA expression in BMDMs. ***p < 0.001. **f**–**j** Studying the TAM-related cancer immunotherapy potential of MAOI treatment in a B16-OVA melanoma syngeneic mouse tumor model. **f** Experimental design. B6 wild-type mice were treated with clodronate liposomes (Clod) to serve as TAM-depleted experimental mice or treated with vehicle liposomes (Veh) to serve as TAM-intact control mice. Phe phenelzine treatment, NT no phenelzine treatment. **g** Tumor growth. **h** Tumor volume at day 18 (***p < 0.001). **i** FACS analyses of CD206 expression on TAMs of TAM-intact experimental mice (*p = 0.0164). **j** FACS analyses of intracellular Granzyme B production in tumor-infiltrating CD8^+^ T cells of all experimental mice (NT, *p = 0.0257; Veh, **p = 0.0025). Veh NT, *n* = 7; Veh Phe, *n* = 8; Clod NT, *n* = 7; Clod Phe, *n* = 7. **k**–**o** Studying the cancer therapy potential of MAOI treatment in combination with anti-PD-1 treatment in the B16-OVA melanoma and MC38 colon cancer syngeneic mouse tumor models (*n* = 5). **k** Experimental design. Tumor-bearing mice were treated with anti-PD-1 antibody (aPD-1) or isotype control (Iso), together with or without phenelzine (Phe) treatment. NT no Phe treatment. **l** B16-OVA tumor growth. **m** B16-OVA tumor volume at day 18. **n** MC38 tumor growth. **o** MC38 tumor volume at day 27. *p < 0.05, **p < 0.01 and ***p < 0.001. Representative of three experiments. Analysed by one-way ANOVA (**b**–**e**, **h**, **j**, **m**, **o**) or by Student’s *t* test (**i**). Statistics are all two-sided. Source data are provided as a Source Data file.

The identification of MAO-A as a key regulator of TAM immunosuppressive polarization makes MAO-A a promising drug target for cancer immunotherapy. Because of the known functions of MAO-A in the brain, small-molecule MAOIs have been developed and clinically utilised for treating various neurological disorders, making it a highly feasible and attractive approach to repurpose these established MAOI drugs for cancer immunotherapy^31,42^. In an in vitro WT BMDM IL-4/IL-13-induced polarization culture (Fig. 5a), addition of multiple MAOIs efficiently reduced ROS levels in BMDMs (Fig. 5b) and suppressed their immunosuppressive polarization, evidenced by their decreased expression of immunosuppressive markers (i.e., CD206; Fig. 5c) and immunosuppressive genes (i.e., *Chi3l3* and *Arg1*; Fig. 5d, e). Notably, the MAOIs that we tested include phenelzine, clorgyline, moclobemide, and pirlindole, covering the major categories of established MAOIs classified on the basis of whether they are non-selective or selective for MAO-A, and whether their effect is reversible (Fig. 5a)^31,43^. Among these MAOIs, phenelzine (trade name: Nardil) is clinically available in the United States^42^. In the following studies, we chose phenelzine as a representative to study the possibility of repurposing MAOIs for cancer immunotherapy, using two syngeneic mouse tumor models: a B16-OVA melanoma model and a MC38 colon cancer model^44^. Of note, phenelzine is a non-selective irreversible MAOI that inhibits both MAO-A and its isoenzyme MAO-B^31^; however, because mouse macrophages predominantly express MAO-A over MAO-B, phenelzine treatment mainly regulates TAM reprogramming via inhibiting MAO-A in these tumor models (Supplementary Fig. 5a).

First, we studied the therapeutic potential of phenelzine in a B16-OVA tumor prevention model (Fig. 5f). Phenelzine treatment effectively suppressed B16-OVA tumor growth in B6 wild-type mice (Fig. 5g, h). No tumor growth difference was observed when we depleted TAMs in experimental mice via a clodronate liposome treatment, indicating that phenelzine suppressed tumor growth via modulating TAMs (Fig. 5g, h and Supplementary Fig. 5b). Correspondingly, TAMs isolated from phenelzine-treated mice displayed a less immunosuppressive phenotype, evidenced by their decreased expression of immunosuppressive markers (i.e., CD206; Fig. 5i) and signature genes (i.e., *Chi3l3* and *Arg1*; Supplementary Fig. 5c) while increased expression of immunostimulatory markers (i.e., CD69, CD86 and I-Ab; Supplementary Fig. 5d–f), that was correlated with an enhanced antitumor reactivity of tumor-infiltrating CD8^+^ T cells (i.e., increased production of Granzyme B; Fig. 5j) in these mice. Further studies showed that phenelzine treatment also effectively suppressed the progression of pre-established solid tumors in both B16-OVA and MC38 models (Supplementary Fig. 5g–k). Notably, similar to that for the B16-OVA tumor model, direct challenge of *Maoa* WT and KO mice with MC38 tumor cells also resulted in a significantly suppressed tumor growth in *Maoa* KO mice, confirming a general impact of MAO-A-deficiency on tumor growth for multiple tumor models (Fig. 1b–d and Supplementary Fig. 5l–n).

Next, we evaluated the potential of phenelzine for combination therapy, in particular combining with other ICB therapies, such as PD-1/PD-L1 blockade therapy (Fig. 5k). Although most ICB therapies target CD8^+^ T cells, these cells are in fact closely regulated by TAMs in the TME, making targeting TAMs another potential avenue for immunotherapy^9,26^. In both B16-OVA and MC38 tumor models, phenelzine treatment significantly suppressed the progression of pre-established solid tumors at a level comparable to the anti-PD-1 treatment; importantly, the combination of phenelzine and anti-PD-1 treatments yielded synergistic tumor suppression efficacy (Fig. 5l–o). These tumor suppression effects of phenelzine were due to immunomodulation but not direct tumor inhibition, because phenelzine treatment did not suppress the growth of B16-OVA and MC38 tumors in immunodeficient NSG mice (Supplementary Fig. 5o–s).

Collectively, these syngeneic mouse tumor model studies provided proof-of-principle evidence for the cancer immunotherapy potential of MAOIs via targeting TAM reprogramming and thereby enhancing antitumor T-cell responses.

### MAO-A blockade for cancer immunotherapy—human TAM and clinical data correlation studies

**Fig. 6:**
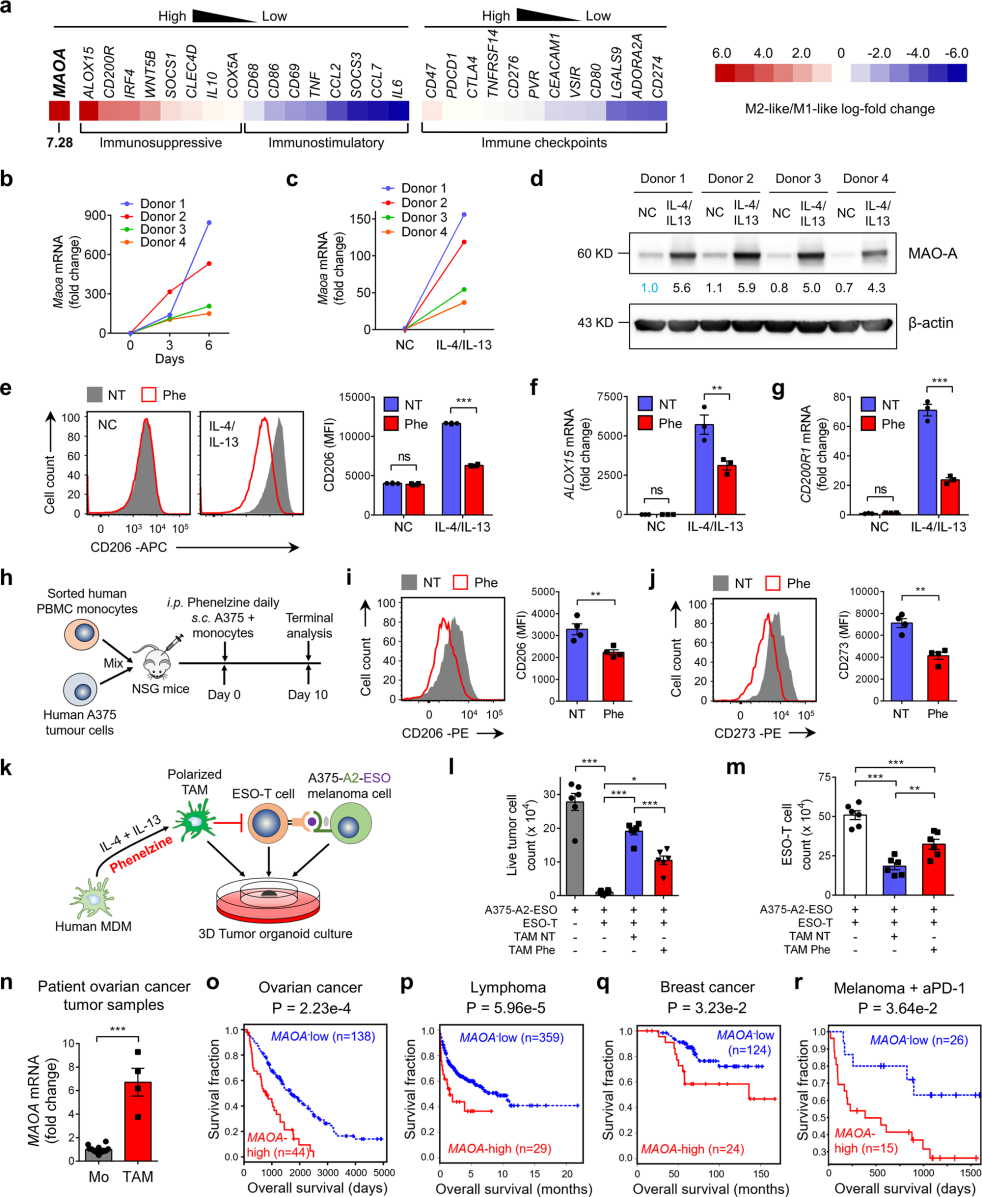
MAO-A blockade for cancer immunotherapy—human TAM and clinical data correlation studies. **a** Heatmap showing the mRNA expression fold change of the indicated genes in human M2-like/M1-like macrophages. **b**–**d** Studying the MAO-A expression in in vitro-cultured human monocyte-derived macrophages (MDMs) (*n* = 4). **b**, **c** QPCR analyses of *MAOA* mRNA expression in MDMs over the 6-day differentiation culture (**b**) and post the IL-4/IL-13-induced polarization (**c**). **d** Western blot analyses of MAO-A protein expression in IL-4/IL-13-polarized MDMs. **e**–**g** Studying the IL-4/IL-13-induced polarization of human MDMs (*n* = 3). **e** FACS analyses of CD206 expression (***p < 0.001). **f**, **g** QPCR analyses of *ALOX15* (**f**) (**p = 0.0012) and *CD200R1* (**g**) (***p < 0.001) mRNA expression. **h**–**j** Studying the in vivo polarization of human macrophages in a human tumor-TAM co-inoculation xenograft mouse model (*n* = 4). **h** Experimental design. **i**, **j** FACS analyses of CD206 (**i**) (**p = 0.0093) and CD273 (**j**) (**p = 0.0013) expression on TAMs (gated as hCD45^+^hCD11b^+^hCD14^+^ cells of TIIs). **k**–**m** Studying the in vitro efficacy of phenelzine in reprogramming human TAMs and enhancing human T-cell antitumor reactivity (*n* = 6). **k** Experimental design. **l**, **m** FACS quantification of live tumor cells (gated as hCD45^−^ cells) and ESO-T cells (gated as hCD45^+^hCD8^+^ESO-TCR^+^ cells). NT no phenelzine treatment. *p < 0.05, **p < 0.01 and ***p < 0.001. **n** QPCR analyses of *MAOA* mRNA expression in human TAMs isolated from ovarian cancer patient tumor samples (*n* = 4). Mo, monocytes isolated from random healthy donor peripheral blood (*n* = 10). ***p < 0.001. **o**–**r** Clinical data correlation studies. Kaplan–Meier plots are presented, showing the association between the intratumoral *MAOA* gene expression levels and overall survival (OS) of cancer patients, in an ovarian cancer patient cohort (GSE26712, *n* = 182; **o**), a lymphoma patient cohort (GSE10846, *n* = 388; **p**), a breast cancers patient cohort (GSE9893, *n* = 148; **q**) and a melanoma patient cohort with anti-PD-1 therapy (PRJEB23709, *n* = 41; **r**). Phe phenelzine, NC no cytokine stimulation, NT no phenelzine treatment. Representative of one (**n**), two (**b**–**d**, **h**–**j**) and three (**e**–**g**, **k**–**m**) experiments. Analysed by one-way ANOVA (**l**, **m**), two-way ANOVA (**e**–**g**), Student’s *t* test (**i**, **j**, **n**), or by two-sided Wald test in a Cox-PH regression (**o**–**r**). Statistics are all two-sided. Source data are provided as a Source Data file.

To explore the translational potential of MAO-A blockade therapy, we first studied MAO-A regulation of human macrophage polarization. Using a Tumor Immune Dysfunction and Exclusion (TIDE) computational method^45^, we analysed the gene expression signatures of in vitro-cultured immunostimulatory M1-like and immunosuppressive M2-like human monocyte-derived macrophages (MDMs) (GSE35449)^46^. Interestingly, among all immune checkpoint, immunostimulatory, and immunosuppressive genes examined, *MAOA* ranked as the top gene with the most dramatically elevated expression in M2-like MDMs (i.e., 7.28 M2/M1 log-fold change; Fig. 6a), suggesting a possible role of MAO-A in promoting human macrophage immunosuppressive polarization. Time-course analysis of MDM culture confirmed an upregulation of MAO-A gene and protein expression during macrophage differentiation that was further upregulated post-IL-4/IL-13-induced immunosuppressive polarization (Fig. 6b–d). Blockade of MAO-A using phenelzine significantly inhibited IL-4/IL-13-induced immunosuppressive polarization of MDMs, evidenced by their decreased expression of immunosuppressive markers (i.e., CD206 and CD273; Fig. 6e and Supplementary Fig. 6a) and signature genes (i.e., *ALOX15* and *CD200R1*; Fig. 6f, g). Collectively, these in vitro data suggest that MAO-A is highly expressed in human macrophages especially during their immunosuppressive polarization, and that MAO-A blockade has the potential to reprogramme human macrophage polarization.

To directly evaluate whether MAOIs could reprogramme human TAM polarization in vivo, we established a human tumor/TAM xenograft NSG mouse model. A375 human melanoma cells were mixed with monocytes sorted from healthy donor peripheral blood mononuclear cells (PBMCs), and s.c. injected into NSG mice to form solid tumors, with or without phenelzine treatment after inoculation (Fig. 6h). Phenelzine treatment effectively suppressed immunosuppressive polarization of human TAMs (gated as hCD45^+^hCD11b^+^hCD14^+^; Supplementary Fig. 6b), supported by their decreased expression of immunosuppressive markers (i.e., CD206 and CD273; Fig. 6i, j).

Next, we studied whether MAOI-induced human TAM reprogramming could impact human T-cell antitumor reactivity, using a 3D human tumor/TAM/T-cell organoid culture (Fig. 6k). NY-ESO-1, a well-recognised tumor antigen commonly expressed in a large variety of human cancers^47^, was chosen as the model tumor antigen. An A375 human melanoma cell line was engineered to co-express NY-ESO-1 as well as its matching MHC molecule, HLA-A2, to serve as the human tumor target (denoted as A375-A2-ESO; Supplementary Fig. 6c, d). NY-ESO-1-specific human CD8^+^ T cells were generated by transducing healthy donor peripheral blood CD8^+^ T cells with a Retro/ESO-TCR retroviral vector encoding a NY-ESO-1-specific TCR (clone 3A1; denoted as ESO-TCR); the resulting T cells, denoted as ESO-T cells, expressed ESO-TCRs and specifically targeted A375-A2-ESO tumor cells, thereby modelling the tumor-specific human CD8^+^ T cells (Supplementary Fig. 6e, f). Human MDMs were cultured from healthy donor PBMCs, followed by IL-4/IL-13 stimulation to induce immunosuppressive polarization in the presence or absence of phenelzine treatment (Fig. 6k). The A375-A2-ESO human melanoma cells, ESO-T cells, and IL-4/IL-13-polarized MDMs were mixed at a 2:2:1 ratio and placed in a 3D tumor organoid culture mimicking TME (Fig. 6k). IL-4/IL-13-polarized MDMs effectively suppressed ESO-T-cell-mediated killing of A375-A2-ESO tumor cells; this immunosuppressive effect was largely alleviated by phenelzine treatment during MDM polarization (Fig. 6l). Accordingly, ESO-T cells co-cultured with phenelzine-treated MDMs, compared to those co-cultured with non-phenelzine-treated MDMs, showed an enhancement in T-cell activation (i.e., increased cell number, increased CD25 expression, and decreased CD62L expression; Fig. 6m and Supplementary Fig. 6g). Collectively, these data suggest that MAOI-induced human TAM reprogramming has the potential to improve antitumor T-cell responses.

To study *MAOA* gene expression in primary human TAMs, we collected fresh ovarian cancer tumor samples from patients, isolated TAMs (sorted as DAPI^−^hCD45^+^hCD11b^+^hTCRαβ^-^hCD14^+^ cells; Supplementary Fig. 6h), and assessed their *MAOA* gene expression. Primary human monocytes isolated from healthy donor PBMCs (sorted as DAPI^−^hCD45^+^hCD11b^+^hTCRαβ^−^hCD14^+^ cells; Supplementary Fig. 6i) were included as controls. Like mouse TAMs, human TAMs expressed high levels of *MAOA* gene, confirming MAO-A as a valid drug target in human TAMs (Figs. 1a and  6n).

Lastly, we conducted clinical data correlation studies to investigate whether intratumoral *MAOA* gene expression is correlated with clinical outcomes in cancer patients, using the TIDE computational method^45^. Intratumoral *MAOA* expression level was negatively correlated with patient survival in multiple cancer patient cohorts spanning ovarian cancer (Fig. 6o)^48^, lymphoma (Fig. 6p)^49^, and breast cancer (Fig. 6q)^50^. Moreover, analysis of a melanoma patient cohort receiving anti-PD-1 treatment showed that high levels of intratumoral *MAOA* expression largely abrogated the survival benefit offered by the PD-1 treatment, suggesting that combining MAO-A blockade therapy with PD-1/PD-L1 blockade therapy may provide synergistic therapeutic benefits through modulating TAM polarization and thereby changing the immunosuppressive TME and improving antitumor immunity (Fig. 6r)^51^. Of note, these whole-tumor lysate transcriptome data analyses could not localise the *MAOA* expression to a specific cell type (e.g., TAMs); future studies of quality transcriptome data generated from single cells or sorted TAMs are needed to obtain such information. Meanwhile, other intratumoral immune cells may also express *MAOA* and can mediate MAO-A-regulated antitumor immunity. For instance, human T cells have been indicated to express *MAOA* (https://www.proteinatlas.org/ENSG00000189221-MAOA/blood); and in our syngeneic mouse tumor model studies using the *Maoa* KO mice, we have detected enhanced antitumor T-cell responses—an effect that may result from a combination of MAO-A’s direct regulation of T cells and indirect regulation of T-cell response via modulating TAMs. The study of MAO-A expression in various intratumoral immune cells, as well as MAO-A regulation of the antitumoral immunity of various immune cells, can be interesting directions for future research. Nonetheless, the present clinical data correlation studies identified MAO-A as a possible negative regulator of survival in a broad range of cancer patients, including those receiving existing ICB therapies, suggesting MAO-A blockade as a promising avenue for developing new forms of cancer therapy and combination therapy.

Taken together, these human TAM and clinical correlation studies confirmed MAO-A as a promising drug target in human TAMs and support the translational potential of MAO-A blockade for cancer immunotherapy through targeting TAM reprogramming.

## Discussion

Based on our findings, we propose an “intratumoral MAO-A-ROS axis” model to elucidate the role of MAO-A in regulating TAM immunosuppressive polarization (Supplementary Fig. 7). Analogous to the well-characterised MAO-A-ROS axis in the brain, where MAO-A controls ROS levels in neurons and thereby modulates neuron degeneration via regulating neuron oxidative stress, the MAO-A-ROS axis in a solid tumor controls ROS levels in TAMs and thereby modulates TAM immunosuppressive polarization via sensitising the IL-4/IL-13-induced JAK-Stat6 signalling pathway (Supplementary Fig. 7). The resemblance between these mechanisms is intriguing: from an evolutionary point of view, it makes sense that some critical molecular regulatory pathways are preserved between the nervous and immune systems, considering that both systems are evolved to defend a living organism by sensing and reacting to environmental danger and stress. Indeed, neurons and immune cells share a broad collection of surface receptors, secretory molecules, and signal transducers^52^. In particular, many neurotransmitters/neuropeptides and their synthesis/degradation machineries traditionally considered specific for neurons are expressed in immune cells, although their functions in the immune system are to a large extent still unknown^53^. Studying these molecules and their regulatory mechanisms may provide new perspectives in tumor immunology and identifying new drug targets for cancer immunotherapies, as exemplified by our current finding of this “MAO-A-ROS axis” regulation of TAM polarization in the TME.

Considering the importance of TAMs in regulating antitumor immunity, there has been considerable efforts in developing cancer therapeutic strategies targeting TAMs. These strategies can be roughly divided into two categories: (1) those which deplete TAMs, and (2) those which alter TAM immunosuppressive activities^26^. The first category includes strategies targeting TAM recruitment and survival, such as blocking the CCL2-CCR2 axis thereby preventing monocyte mobilisation from the bone marrow and recruitment into inflammatory sites, or blocking the CSF1-CSF1R axis thereby inducing apoptosis of TAMs, or blocking the CXCL12-CXCR4 and angiopoietin 2 (ANG2)-TIE2 axes thereby depleting TIE2^+^ macrophages that are critical for tumor angiogenesis^26,54^. However, an intrinsic downside of depleting TAMs is the loss of their innate immunostimulatory role as the primary phagocytes and professional antigen-presenting cells (APCs) in solid tumors. Reprogramming or repolarizing immunosuppressive TAMs towards an immunostimulatory phenotype therefore can be an attractive direction; this second category of TAM-repolarizing strategies includes those reprogramming TAMs via CD40 agonists, HDAC inhibitors, PI3Kγ inhibitors, and creatine^26,55–58^. Many of these TAM reprogramming strategies are currently under active clinical evaluation^26^. Notably, CD40 agonists work through activating CD40L-downstream NF-kB pathway^56,59^; HDAC inhibitors work through altering histone modifications^55,60^; PI3Kγ inhibitors work through stimulating NF-κB activation while inhibiting C/EBPβ activation^57,61^; and creatine uptake works through regulating cytokine responses^58^. Our discovery of MAO-A as a critical regulator of TAM polarization through modulating oxidative stress provides a drug target and a mechanism of action (MOA) for expanding TAM-repolarizing strategies.

Compared to many new therapeutic candidates, MAO-A is unique in that it is already an established drug target due to its known functions in the brain^42^. In fact, small-molecule MAOIs have been developed to block MAO-A enzymatic activity in the brain and are clinically used for treating various neurological disorders^42^. Notably, some MAOIs cross-inhibit the MAO-A isoenzyme MAO-B, that co-expressed with MAO-A in the brain (Supplementary Fig. 7)^31^. However, in human macrophages, especially in M2-like immunosuppressive macrophages, MAO-A is the dominant form (i.e., the expression of *MAOA* was about 40-fold higher than that of *MAOB* in M2-like human macrophages; Supplementary Fig. 8)^46^. Of course, other tissues and cells may express significant levels of MAO-B that is subjected to MAOI inhibition and may mediate part of the MAOI-induced therapeutic effects. In our studies, we tested multiple clinically approved MAOIs (phenelzine, clorgyline, moclobemide, and pirlindole) and demonstrated their efficacy in regulating macrophage ROS levels and immunosuppressive polarization, pointing to the possibility of repurposing these drugs for cancer immunotherapy (Figs. 5 and 6). Developing new cancer drugs is extremely costly and time-consuming; drug repurposing offers an economic and speedy pathway to novel cancer therapies because approved drugs have known safety profiles and modes of actions and thus can enter the clinic quickly^62^.

MAOIs had been used extensively over two decades after their introduction in the 1950s, but since then their use has declined because of reported side effects and the introduction of other classes of antidepressant drugs^42^. However, these MAOI side effects may be manageable. For instance, a claimed major side effect of MAOIs is the risk of triggering tyramine-induced hypertensive crisis when patients eat tyramine-rich foods such as aged cheese (hence, “cheese effects”), which has led to cumbersome food restrictions^42^. The development of reversible and increasingly MAO-B-selective MAOI agents administered via a transdermal delivery system (i.e., the EMSAM selegiline transdermal system) has largely avoided the tyramine related “cheese effects” and thereby can relieve food restrictions and improve the overall safety of MAOIs^63^. Interest in MAOIs as a major class of antidepressants is reviving, and repurposing MAOIs for cancer immunotherapy can be an attractive application of these potent drugs^42^. Moreover, many cancer patients suffer from depression and anxiety; these overwhelming emotional changes can negatively interfere with the quality of life and cancer treatment efficacy of cancer patients^64^. Repurposing MAOIs for cancer immunotherapy thus may provide cancer patients with anti-depression and antitumor dual benefits, making this therapeutic strategy particularly attractive. Nonetheless, caution about drug:food and drug:drug interactions are still relevant for MAOIs’ cancer therapy application, evidenced by the side effects (e.g., hypertension) observed in a recent Phase 2 trial of MAOI phenelzine in biochemical recurrent prostate cancer^65^.

Because preclinical evidence largely supports combinatorial approaches being necessary to achieve significant antitumor efficacy, most TAM-targeting strategies currently under clinical evaluation are tested in combination with standard chemotherapy or radiation therapy or in combination with T-cell-directed ICB therapies such as PD-1 or/and PD-L1 blockade therapy^26^. In our study, we found that MAOI treatment synergised with anti-PD-1 treatment in suppressing syngeneic mouse tumor growth (Fig. 5k–o), and that intratumoral *MAOA* gene expression levels dictated poor patient survival in melanoma patients receiving anti-PD-1 therapy (Fig. 6r). These data highlight the promise of MAOI treatment as a valuable component for combination cancer therapies.

Interestingly, MAO-A upregulation has been detected in cancerous tissues compared to normal tissues and MAO-A expression has been associated with cancer metastases and decreased cancer-related patient survival for several cancers, including prostate cancer^66,67^, lung cancer^68,69^, breast cancer^70^, glioma^71^ and lymphoma^72^. Together with our finding, these studies suggest the possible multifaceted function of MAO-A in promoting certain cancers, through the direct promotion of tumor development and indirect suppression of antitumor immunity. Consequently, MAO-A blockade therapy may provide dual therapeutic benefits for these cancers, by both suppressing tumor metastasis and enhancing antitumor immunity.

In summary, here we identified MAO-A as a critical molecule regulating TAM immunosuppressive polarization and thereby modulating antitumor immunity, and demonstrated the potential of repurposing established MAOI antidepressants for cancer immunotherapy. Future clinical studies are encouraged to investigate the clinical correlations between MAOI treatment and clinical outcomes in cancer patients and to explore the possibility of repurposing MAOIs for combination cancer therapies. Meanwhile, the immune regulatory function of MAO-A certainly goes beyond regulating TAM polarization. Notably, in another recent study, we have identified MAO-A as an immune checkpoint restraining antitumor T-cell immunity through controlling intratumoral T-cell autocrine serotonin signalling^73^. MAO-A’s capacity to regulate various components of antitumor immunity is attractive, suggesting that MAO-A blockade may be multifunctional for cancer immunotherapy. It is also likely that MAO-A regulates immune reactions to other diseases such as infectious diseases and autoimmune diseases. Studying the roles of MAO-A in regulating various immune cells under different health and disease conditions will be interesting topics for future research.

## Methods

### Mice

C57BL/6J (B6), B6.SJL-*Ptprc*^*a*^*Pepc*^*b*^/BoyJ (CD45.1, BoyJ), 129S-*Maoa*^*tm1Shih*^/J (*Maoa* KO)^33^ and NOD.Cg-*Prkdc*^*scid*^ *Il2rg*^*tm1Wjl*^/SzJ (NSG) mice were purchased from the Jackson Laboratory (Bar Harbor). *Maoa* KO mice were backcrossed with C57BL/6J mice for more than nine generations at the University of California, Los Angeles (UCLA). Eight- to twelve-week-old female mice were used for all experiments unless otherwise indicated. Due to ethical reasons, we ended experiments before tumor volume surpassed 1000 mm^3^. All mice experiments were repeated at least three times unless specifically mentioned. Replicates of each individual experiment are stated in its figure legends. All animals were maintained at the UCLA animal facilities and all animal experiments have complied with all relevant ethical regulations approved by the Institutional Animal Care and Use Committee of UCLA.

### Human tumor samples

All human tumor samples were obtained following institutional guidelines under protocols approved by the institutional review boards (IRBs) at the UCLA Medical Center. Primary human ovarian cancer tumor samples were obtained from the operating room at the UCLA Medical Center from consenting patients and experiments have complied with all relevant ethical regulations using IRB-approved protocols (IRB# 10-000727). Tumors specimens were brought back to the laboratory for further analyses. Detailed samples information is provided in Supplementary Table 1, including diagnosis and staging.

### Cell lines and viral vectors

The B16-OVA mouse melanoma cell line and the PG13 retroviral packaging cell line were provided by Dr. Pin Wang (University of Southern California, CA)^74^. The MC38 mouse colon adenocarcinoma cell line was provided by M. Bosenberg (Yale)^44^. The HEK 293T and Phoenix-ECO retroviral packaging cell lines, the A375 human melanoma cell line, and the L929 mouse connective tissue cell line were purchased from the American Type Culture Collection (ATCC). The A375-A2-ESO cell line was previously reported^75^. The Phoenix-ECO-MIG, Phoenix-ECO-MIG-*Maoa*, and PG13-ESO-TCR stable virus-producing cell lines were generated in this study. The MIG (MSCV-IRES-GFP) retroviral vector was reported previously^76–78^. MIG-*Maoa* and Retro/ESO-TCR retroviral vectors were generated in this study.

### Syngeneic mouse tumor models

B16-OVA melanoma cells (1 × 10^6^ per animal) or MC38 colon cancer cells (5 × 10^5^ per animal) were subcutaneously (s.c.) injected into experimental mice to form solid tumors. In some experiments, mice received an intraperitoneal (i.p.) injection of phenelzine (30 mg/kg/day) to block MAO-A activity. In some experiments, mice received i.p. injection of clodronate liposomes (200 μl/animal, twice per week) to deplete TAMs; mice received i.p. injection of vehicle liposomes (200 μl/animal, twice per week) were included as controls. In some experiments, mice received i.p. injection of anti-mouse PD-1 antibodies (300 μg/animal, twice per week) to block PD-1; mice received i.p. injection of isotype antibodies was included as controls. During an experiment, tumor growth was monitored twice per week by measuring tumor size using a Fisherbrand^TM^ Traceable^TM^ digital caliper (Thermo Fisher Scientific); tumor volumes were calculated by formula 1/2 × L × W^2^. At the end of an experiment, solid tumors were collected and tumor-infiltrating immune cells were isolated for analysis using QPCR, flow cytometry, and/or scRNASeq.

### Bone marrow (BM) transfer mouse tumor model

BM cells were collected from femurs and tibias of *Maoa* WT and *Maoa* KO donor mice, and were separately transferred into BoyJ (CD45.1) wild-type recipient mice that were preconditioned with whole-body irradiation (1200 rads). Recipient mice were maintained on antibiotic water (Amoxil, 0.25 mg/ml) for 4 weeks after BM transplantation. Periodical bleedings were performed to monitor immune cell reconstitution using flow cytometry. Tumor inoculation started at 12 weeks post BM transfer when recipient mice were fully immune reconstituted. B16-OVA mouse melanoma cells were s.c. injected into recipient mice to form solid tumors (1 × 10^6^ cells per animal). Tumor growth was monitored twice per week by measuring tumor size using a Fisherbrand^TM^ Traceable^TM^ digital caliper; tumor volumes were calculated by formula 1/2 × L × W^2^. At the end of an experiment, tumor-infiltrating immune cells were isolated for analysis using flow cytometry.

### Syngeneic mouse tumor-TAM co-inoculation model

Bone marrow cells were collected from *Maoa* WT and *Maoa* KO mice and were cultured in vitro to generate bone marrow-derived macrophages (BMDMs). B16-OVA tumor cells (1 × 10^6^ cells per mouse) and BMDMs (5 × 10^6^ cells per mouse) were mixed and s.c. injected into BoyJ mice to form solid tumors. Tumor growth was monitored twice per week by measuring tumor size using a Fisherbrand^TM^ Traceable^TM^ digital calliper; tumor volumes were calculated by formula 1/2 × L × W^2^. At the end of an experiment, tumors were collected and tumor-infiltrating immune cells were isolated for analysis using flow cytometry.

### Xenograft human tumor-TAM co-inoculation model

Human peripheral blood mononuclear cells (PBMCs) of healthy donors were obtained from the CFAR Gene and Cellular Therapy Core Laboratory at UCLA, without identification information under federal and state regulations. Human monocytes were isolated from healthy donor PBMCs via magnetic-activated cell sorting (MACS) using human CD14 microbeads (Miltenyi Biotec, 130-050-201) followed by fluorescence-activated cell sorting (FACS; sorted as hCD45^+^hCD11b^+^hCD14^+^ cells) using a FACSAria II flow cytometer (BD Biosciences). Human A375 melanoma cells (10 × 10^6^ cells per animal) and purified human monocytes (5 × 10^6^ cells per animal) were mixed and s.c. injected into NSG mice to form solid tumors. Some experimental animals received i.p. injection of MAOI (phenelzine, 30 mg/kg/day) to block MAO-A activity. At the end of an experiment, tumor-associated immune cells were isolated for analysis using flow cytometry.

### Tumor-infiltrating immune cell (TII) isolation and analysis

Solid tumors were collected from experimental mice at the termination of a tumor experiment. Tumors were cut into small pieces and smashed against a 70-μm cell strainer (Corning, 07-201-431) to prepare single cells. Immune cells were enriched through gradient centrifugation with 45% Percoll (Sigma-Aldrich, P4937) at 800×*g* for 30 min at 25 °C without braking, followed by treatment with Tris-buffered ammonium chloride buffer to lyse red blood cells according to a standard protocol (Cold Spring Harbor Protocols). The resulting TII isolates were then used for further analysis.

In some experiments, TII isolates were sorted via FACS using a FACSAria II flow cytometer (BD Biosciences) to purify TAMs (sorted as DAPI^−^CD45.2^+^CD11b^+^Ly6G^−^Ly6C^−/low^F4/80^+^ cells), which were then subjected to QPCR analysis of *Maoa* mRNA expression in TAMs.

In some experiments, TII isolates were sorted via FACS using a FACSAria II flow cytometer (BD Biosciences) to purify immune cells (sorted as DAPI^−^CD45.2^+^ cells), which were then subjected to scRNASeq analysis of gene expression profiling of TIIs.

In some experiments, TII isolates were directly analysed using MACSQuant Analyzer 10 Flow Cytometer (Miltenyi Biotec) to study the cell surface marker expression of TAMs (pre-gated as CD45.2^+^CD11b^+^Ly6G^−^Ly6C^−/low^F4/80^+^ cells) and the intracellular effector molecule production of CD8^+^ T cells (pre-gated as CD45.2^+^TCRβ^+^CD8^+^ cells).

### Mouse monocyte isolation

Peripheral blood samples collected from experimental mice were treated with Tris-buffered ammonium chloride buffer to lyse red blood cells according to a standard protocol (Cold Spring Harbor Protocols). The resulting mononuclear cells were sorted via FACS using a FACSAria II flow cytometer (BD Biosciences) to purify monocytes (sorted as DAPI^−^CD45.2^+^CD11b^+^Ly6G^−^Ly6C^+^ cells), which were then subjected to QPCR analysis of *Maoa* mRNA expression.

### Mouse bone marrow-derived macrophages (BMDM) culture and polarization

To generate BMDMs, BM cells were collected from femurs and tibias of *Maoa* WT mice and *Maoa* KO mice, and were cultured in C10 medium containing 20% of L929-conditional medium in a 10-cm dish (2 × 10^6^ cells per ml; 12 ml per dish) for 6 days. At day 6, the resulting BMDMs were collected and reseeded in a six-well plate (1 × 10^6^ cells per ml; 2 ml per well) in C10 medium for 24 h, in the presence or absence of recombinant murine IL-4 (10 ng/ml) (Peprotech, 200-04) and IL-13 (10 ng/ml) (Peprotech, 200-13) to induce BMDM immunosuppressive polarization.

In some experiments, MAOIs were added to the *Maoa* WT BMDM polarization culture 30 min prior to adding recombinant murine IL-4 and IL-13, to block MAO-A activity during BMDM polarization. MAOIs studied were phenelzine (Phe, 20 μM) (Sigma-Aldrich), clorgyline (Clo, 20 μM) (Sigma-Aldrich), moclobemide (Moc, 200 μM) (Sigma-Aldrich), and pirlindole (Pir, 20 μM) (R&D Systems). At 24 h after IL-4/IL-13 stimulation, BMDMs were collected for analysis.

In some experiments, H_2_O_2_ (100 μM) were added to the *Maoa* WT and *Maoa* KO BMDM polarization culture 30 min prior to adding recombinant murine IL-4 and IL-13. At 30 min after IL-4/IL-13 stimulation, BMDMs were collected for WB analysis; at 24 h after IL-4/IL-13 stimulation, BMDMs were collected for flow cytometry and QPCR analysis.

In some experiments, tyramine (100 μM) (Sigma-Aldrich, T90344) was added to the *Maoa* WT and *Maoa* KO BMDM polarization culture 30 min prior to adding recombinant murine IL-4 and IL-13. At 24 h after IL-4/IL-13 stimulation, BMDMs were collected for flow cytometry and QPCR analysis.

### Macrophage suppressive function assay

IL-4/IL-13 polarized *Maoa* WT and *Maoa* KO BMDMs were mixed with splenocytes harvested from B6 wild-type mice at 0:1, 1:2, 1:4 or 1:8 ratio, then cultured in a 24-well plate in C10 medium (1 × 10^6^ splenocytes/ml/well), in the presence of plate-bound anti-mouse CD3ε (5 μg/ml) and soluble anti-mouse CD28 (1 μg/ml) for 2 days. At the end of a culture, cells were collected for flow cytometry analysis.

### MIG-*Maoa* retroviral vector construction, production, and macrophage transduction

MIG retroviral vector was reported previously^76–78^. Codon-optimised *Maoa* cDNA (synthesised by IDT) was inserted into a MIG retroviral vector to generate the MIG-*Maoa* retroviral vector. Vsv-g-pseudotyped MIG and MIG-*Maoa* retroviruses were produced using HEK 293T virus packaging cells following a standard calcium precipitation method^77,78^, and then were used to transduce Phoenix-ECO cells to generate stable cell lines producing ECO-pseudotyped MIG or MIG-*Maoa* retroviruses (denoted as Phoenix-ECO-MIG and Phoenix-ECO-MIG-*Maoa* cell lines, respectively). For virus production, Phoenix-ECO-MIG and Phoenix-ECO-MIG-*Maoa* cells were seeded at a density of 0.8 × 10^6^ cells per ml in D10 medium, and cultured in a 15-cm dish (30 ml per dish) for 2 days. Virus supernatants were then collected and used for macrophage transduction.

BM cells harvested from *Maoa* WT and *Maoa* KO mice were cultured in a six-well plate in C10 medium containing 20% L929-conditional medium (4 × 10^6^ cells/2 ml/well) for 6 days, to differentiate into BMDMs. From day 1 to day 5, cells were spin-infected daily with virus supernatants supplemented with polybrene (10 μg/ml) at 660×*g* at 30 °C for 90 min. At day 6, recombinant murine IL-4 (10 ng/ml) and IL-13 (10 ng/ml) were added to cell culture to induce BMDM immunosuppressive polarization. At day 7, transduced BMDMs were collected for flow cytometry analysis of transduction efficiency (%GFP^+^ cells of total cells); GFP^+^ BMDMs were sorted via FACS using a FACSAria II flow cytometer (BD Biosciences) and were then used for QPCR analysis of immunosuppressive gene expression.

### Human monocyte-derived macrophage (MDM) culture and polarization

Human peripheral blood mononuclear cells (PBMCs) of healthy donors were obtained from the CFAR Gene and Cellular Therapy Core Laboratory at UCLA, without identification information under federal and state regulations. Human monocytes were isolated from healthy donor PBMCs by adherence. Briefly, PBMCs were suspended in serum-free RPMI 1640 media (Corning Cellgro, 10-040-CV) at 10 × 10^6^ cells/ml. In total, 12.5 ml of the cell suspension were added to each 10-cm dish and incubated for an hour in a humidified 37 °C, 5% CO_2_ incubator. Medium that contained non-adherent cells was discarded. Dishes were washed twice and adherent monocytes were cultured in C10 media with human M-CSF (10 ng/ml) (Peprotech, 300-25) for 6 days to generate MDMs. At day 6, the resulting MDMs were collected and reseeded in a 6-well plate in C10 medium (1 × 10^6^ cells/ 2 ml/well) for 48 h, in the presence or absence of recombinant human IL-4 (10 ng/ml) (Peprotech, 214-14) and human IL-13 (10 ng/ml) (Peprotech, 214-13) to induce MDM immunosuppressive polarization. In some experiments, MAOIs (phenelzine, 20 μM) were added to the MDM polarization culture 30 min prior to adding recombinant human IL-4 and human IL-13, to block MAO-A activity during MDM polarization. Polarized MDMs were then collected and used for flow cytometry and QPCR analysis or for setting up the 3D human tumor organoid culture experiments.

### Human NY-ESO-1-specific TCR-engineered CD8^+ ^T (ESO-T) cells

The Retro/ESO-TCR vector was constructed by inserting into the parental pMSGV vector a synthetic gene encoding an HLA-A2-restricted, NY-ESO-1 tumor antigen-specific human CD8 TCR (clone 3A1)^75^. Vsv-g-pseudotyped Retro/ESO-TCR retroviruses were generated by transfecting HEK 293T cells following a standard calcium precipitation protocol and an ultracentrifugation concentration protocol; the viruses were then used to transduce PG13 cells to generate a stable retroviral packaging cell line producing GALV-pseudotyped Retro/ESO-TCR retroviruses (denoted as the PG13-ESO-TCR cell line). For virus production, the PG13-ESO-TCR cells were seeded at a density of 0.8 × 10^6^ cells per ml in D10 medium, and cultured in a 15-cm dish (30 ml per dish) for 2 days; virus supernatants were then harvested and stored at −80 °C for future use.

Healthy donor PBMCs were cultured in a 12-well plate in C10 medium (1 × 10^6^ cells/ml/well) for 2 days, stimulated with Dynabeads™ Human T-Activator CD3/CD28 (10 μl/ml) (GIBCO, 11161D) and recombinant human IL-2 (20 ng/ml) (Peprotech). After 2 days, dynabeads were removed and cells were spin-infected with frozen-thawed Retro/ESO-TCR retroviral supernatants supplemented with polybrene (10 μg/ml) at 660×*g* at 30 °C for 90 min following an established protocol^75^. Transduced human CD8^+^ T cells (denoted as ESO-T cells) were expanded for another 6–8 days in C10 medium containing recombinant human IL-2 (20 ng/ml) (Peprotech) and then cryopreserved for future use. Mock-transduced human CD8^+^ T cells (denoted as Mock-T cells) were generated as controls.

### 3D human tumor/TAM/T-cell organoid culture

A375-A2-ESO human melanoma cell line was generated by engineering the parental A375 cell line to overexpress a NY-ESO-1 tumor antigen as well as its matching HLA-A2 molecule^75^. Human MDMs were generated from healthy donor PBMCs and polarized with IL-4/IL-13 in the presence or absence of phenelzine treatment. ESO-T cells were generated by engineering healthy donor PBMC CD8^+^ T cells to express a NY-ESO-1-specific TCR (clone 3A1). The A375-A2-ESO tumor cells, MDMs, and ESO-T cells were mixed at a 2:1:2 ratio. Mixed cells were centrifuged and resuspended in C10 medium at 1 × 10^5^ cells per μl medium. The cell slurry was adjusted to 5 μl per aggregate and was gently transferred onto a microporous membrane cell insert (Millicell, PICM0RG50) using a 20-μl pipet to form a 3D human tumor/TAM/T-cell organoid. Prior to cell transfer, cell inserts were placed in a six-well plate immersed with 1 ml C10 medium. Two days later, the organoids were dissociated by P1000 pipet tip and disrupted through a 70-μm nylon strainer to generate single-cell suspensions for further analysis.

### Reagents

Adherent cell line culture medium (denoted as D10 medium) was made of Dulbecco’s modified Eagle’s medium (DMEM, Corning Cellgro, 10-013-CV) supplemented with 10% fetal bovine serum (FBS, Sigma-Aldrich, F2442) and 1% penicillin–streptomycin–glutamine (Gibco, 10378016). T-cell and macrophage culture medium (denoted as C10 medium) was made of RPMI 1640 (Corning Cellgro, 10-040-CV) supplemented with 10% FBS (Sigma-Aldrich), 1% penicillin–streptomycin–glutamine (Gibco), 0.2% normocin (Invivogen, ant-nr-2), 1% MEM non-essential amino acids solution (Gibco, 11140050), 1% HEPES (Gibco, 15630056), and 1% sodium pyruvate (Gibco, 11360070).

Macrophage culture reagents, including recombinant murine IL-4, recombinant murine IL-13, recombinant human M-CSF, recombinant human IL-4, and recombinant human IL-13, were purchased from PeproTech. T-cell culture reagents, including purified NA/LE anti-mouse CD3ε (clone 145-2C11), anti-mouse CD28 (clone 37.51), anti-human CD3 (clone OKT3), and anti-human CD28 (clone CD28.2), were purchased from BD Biosciences. Recombinant human IL-2 was purchased from PeproTech. Hydrogen peroxide solution was purchased from Sigma-Aldrich (216763).

In vivo PD-1 blocking antibody (clone RMP1-14) and its isotype control (rat IgG2a) were purchased from BioXCell. In vivo TAM depletion clodronate liposomes and their control vehicle liposomes were purchased from Clodrosome.

Monoamine oxidase inhibitors (MAOIs), including phenelzine, moclobemide and clorgyline, were purchased from Sigma-Aldrich. Pirlindole was purchased from R&D systems.

### Flow cytometry

Flow cytometry, also known as FACS (fluorescence-activated cell sorting), was used to analyse surface marker and intracellular effector molecule expression in immune cells. Fluorochrome-conjugated monoclonal antibodies specific for mouse CD45.2 (clone 104), CD11b (Clone M1/70), Ly6G (Clone 1A8), F4/80 (Clone BM8), Ly6C (Clone HK1.4), CD206 (Clone C068C2), CD69 (clone H1.2F3), CD86 (Clone GL-1), I-Ab (Clone AF6-120.1), TCRβ (clone H57-597), CD45.1 (Clone A20), CD4 (Clone GK1.5), CD8 (clone 53-6.7), CD25 (clone PC61), CD44 (clone IM7), CD62L (clone MEL-14) and Granzyme B (Clone QA16A02) were purchased from BioLegend. Mouse Fc Block (anti-mouse CD16/32; clone 2.4G2) was purchased from BD Biosciences. Fluorochrome-conjugated monoclonal antibodies specific for human CD45 (clone H130), CD11b (Clone ICRF44), CD14 (Clone HCD14), CD206 (Clone 15-2), CD273 (Clone 24 F.10C12), TCRαβ (clone I26), CD4 (clone OKT4), CD8 (clone SK1), CD44 (clone IM7), CD62L (clone DREG-56), and human Fc Receptor Blocking Solution (TruStain FcX™, 422302) were purchased from BioLegend. Fixable Viability Dye eFluor 506 was purchased from Thermo Fisher Scientific. DAPI (Thermo Fisher Scientific) was included to exclude dead cells in FACS sorting.

To study cell surface marker expression, cells were stained with Fixable Viability Dye followed by Fc blocking and surface marker staining, following a standard procedure as described previously^78^. To study T-cell intracellular cytotoxicity molecule production, intracellular staining of Granzyme B was performed using the BD Cytofix/CytopermTM Fixation/Permeabilization Kit (BD Biosciences, 55474) following the manufacturer’s instructions. These cells were co-stained with surface markers to identify CD8^+^ T cells (gated as TCRβ^+^CD8^+^ cells in vitro or CD45.2^+^TCRβ^+^CD8^+^ cells in vivo).

Stained cells were analysed using a MACSQuant Analyzer 10 flow cytometer (Miltenyi Biotec); data were analysed using a FlowJo software (BD Biosciences).

Detailed reagent information is provided in Supplementary Table 2.

### Western blot (WB)

Total protein was extracted using a RIPA lysis buffer (PIERCE, Roche, Thermo Fisher Scientific) supplemented with protease inhibitor cocktail cOmplete Mini (one tablet/10 ml) (Sigma-Aldrich, 4693159001) and phosphatase inhibitor PhosSTOP (one tablet/10 ml) (Sigma-Aldrich, 4906845001), then transferred to pre-cooled Eppendorf tubes. The lysed solution was kept on ice for 30 min and then centrifuged at 15,000×*g* for 5 min at 4 °C. Supernatants were collected and protein concentrations were quantified using a BCA protein assay (PIERCE, Thermo Fisher Scientific, 23225). Equal amounts of protein were loaded and separated by 8% sodium dodecyl sulfate-polyacrylamide gel electrophoresis (SDS-PAGE), and then transferred to an Immunobilon-P PVDF Membrane (Millipore). The membranes were blocked with a SuperBlock™ T20 (TBS) Blocking Buffer (Thermo Fisher Scientific, 37536). Antibodies were diluted in 5% nonfat milk dissolved in washing buffer TBST (20 mM Tris–HCl, 150 mM NaCl, 0.1% Tween-20).

Primary antibodies against mouse Stat6, p-Stat6 (Tyr641), JAK1, p-JAK1 (Tyr1034/1035), JAK2, p-JAK2 (Tyr1008), JAK3, p-JAK3 (Tyr980/981), HRP-labelled anti-rabbit secondary antibodies, and HRP-labelled anti-mouse secondary antibodies were purchased from Cell Signaling Technology. MAO-A antibody was purchased from Abcam (Clone EPR7101). Primary antibodies against β-actin (Santa Cruz Biotechnology) were used as an internal control for total protein extracts. Signals were visualised using a ChemiDoc Image System (Bio-Rad). Data were analysed using Image J software (Bio-Rad). Signal intensity was normalised using housekeeping protein and compared to a sample labelled in azure in each row.

Detailed reagent information is provided in Supplementary Table 2.

### Quantitative real-time PCR (QPCR)

The total RNA was extracted from cells using TRIzol reagent (Invitrogen, Thermo Fisher Scientific, 15596018) following the manufacturer’s instructions. SuperScript III First-strand (Thermo Fisher Scientific, 18080051) was used for reverse transcription. QPCR was performed using a KAPA SYBR FAST qPCR Master Mix (Kapa Biosystems) and a 7500 Real-time PCR System (Applied Biosystems) according to the manufacturers’ instructions. Housekeeping gene *Ube2d2* was used as an internal control for mouse immune cells and *ACTB* was used as an internal control for human immune cells. The relative expression of a target gene was calculated using the 2^ΔΔCT^ method. All primers used in this study are listed in Supplementary Table 3.

### Reactive oxygen species (ROS) measurement

Cells were stained with surface marker antibodies, washed with PBS, then resuspended in pre-warmed PBS (1 × 10^6^ cells/ml/tube) containing 1 µM CM-H2DCFDA (Thermo Fisher Scientific, C6827). After 15 min of incubation at room temperature, cells were immediately washed with cold PBS followed by flow cytometry analysis. ROS levels were measured by oxidation of the CM-H2DCFDA probes that can be read out as the fluorescence intensity at the FITC/488 channel of a flow cytometer.

### Single-cell RNA sequencing (scRNAseq)

scRNASeq was used to analyse the gene expression profiles of TIIs. B16-OVA tumors were harvested from *Maoa* WT and *Maoa* KO mice to prepare TII suspensions (ten tumors were combined for each group). TII suspensions were then sorted using a FACSAria II flow cytometer to purify immune cells (gated as DAPI^−^CD45.2^+^ cells). Sorted TIIs were immediately delivered to the Technology Center for Genomics & Bioinformatics (TCGB) facility at UCLA for library construction and sequencing. Cells were stained with trypan blue (Thermo Fisher Scientific, T10282) and counted using a Cell Countess II automated cell counter (Thermo Fisher Scientific). 10,000 TIIs from each experimental group were loaded on the Chromium platform (10X Genomics) and libraries were constructed using a Chromium Single Cell 3’ library & Gel Bead Kit V2 (10X Genomics, PN-120237) according to the manufacturer’s instructions. Libraries were sequenced on an Illumina Novaseq 6000 System, using a Novaseq 6000 S2 Reagent Kit (100 cycles; 20012862, Illumina). Data analysis was performed using a Cellranger Software Suite (10X Genomics). BCL files were extracted from the sequencer and used as inputs for the cellranger pipeline to generate the digital expression matrix for each sample. Then cellranger aggr command was used to aggregate the two samples into one digital expression matrix. The matrix was analysed using Seurat, an R package designed for single-cell RNA sequencing. Specifically, cells were first filtered to have at least 300 UMIs (unique molecular identifiers), at least 100 genes, and at most 5% mitochondrial gene expression; only one cell did not pass the filter. The filtered matrix was normalised using the Seurat function NormalizeData. Variable genes were found using the Seurat function FindVariableGenes. The matrix was scaled to regress out the sequencing depth for each cell. Variable genes that had been previously identified were used in principle component analysis (PCA) to reduce the dimensions of the data. Following this, 13 PCs were used in UMAP to further reduce the dimensions to 2. The same 13 PCs were also used to group the cells into different clusters by the Seurat function FindClusters. Next, marker genes were found for each cluster and used to define the cell types. Subsequently, two clusters of TAMs (identified by co-expression of *Mrc1* and *Cd86* signature genes) were extracted and compared between the *Maoa* WT and *Maoa KO* samples. Expression distribution of immunosuppressive and immunostimulatory signature genes in *Maoa* WT and *Maoa* KO TAMs were compared and presented in violin plots. scRNAseq datasets have been uploaded to GEO database with an accession number GSE153615.

### Tumor immune dysfunction and exclusion (TIDE) computational method

TIDE analyses were conducted as previously described (http://tide.dfci.harvard.edu)^45^. Two functions of the TIDE computational method were used: (1) the prioritisation function and (2) the survival correlation function.

The prioritisation function of TIDE was used to rank a target gene by its immune dysfunction/risk score, which for TAMs, was calculated as its gene expression log-fold change of M2-like/M1-like MDMs^45^. A transcriptome dataset (GSE35449) was used, which was generated by microarray analysis of the gene expression profiling of in vitro polarized M1-like or M2-like human MDMs^46^. A score higher than 1 indicates the preferential expression of a gene in M2-like compared to M1-like human macrophages. The higher a score is, the more “prioritised” a gene is in relating to TAM immunosuppressive polarization.

The survival correlation function of TIDE was used to study the clinical data correlation between the intratumoral *MAOA* gene expression and patient survival. Four patient cohorts were analysed: ovarian cancer (GSE26712)^48^, lymphoma (GSE10846)^49^, breast cancer (GSE9893)^50^, and melanoma (PRJEB23709)^51^. For each patient cohort, tumor samples were divided into two groups: *MAOA*-high (samples with *MAOA* expression one standard deviation above the average) and *MAOA*-low (remaining samples) groups. The association between the intratumoral *MAOA* gene expression levels and patient overall survival (OS) was computed through the two-sided Wald test in the Cox-PH regression and presented in Kaplan–Meier plots. *p* value indicates the comparison between the *MAOA*-low and *MAOA*-high groups and was calculated by two-sided Wald test in a Cox-PH regression.

### Statistical analysis

GraphPad Prism 6 (GraphPad Software) was used for the graphic representation and statistical analysis of the data. All data were presented as the mean ± standard error of the mean (SEM). A two-tailed Student’s *t* test was used for comparison between groups. Multiple comparisons were performed using an ordinary one-way ANOVA followed by Tukey’s multiple comparisons test or using a two-way ANOVA followed by Sidak’s multiple comparisons test. *p* < 0.05 was considered statistically significant. ns not significant; *p < 0.05; **p < 0.01; ***p < 0.001. For scRNAseq data analysis, Wilcoxon rank-sum test was utilised to determine the *p* value between the two groups. Benjamini–Hochberg Procedure was used to adjust the *p* value to reduce the false-positive rate. For the Kaplan–Meier plot of the overall patient survival for ovarian cancer, lymphoma, breast cancer, and melanoma with different *MAOA* levels, the *p* value was calculated by two-sided Wald test in a Cox-PH regression.

### Reporting summary

Further information on research design is available in the Nature Research Reporting Summary linked to this article.

## Supplementary information

Supplementary Information

Reporting Summary

## Data Availability

The scRNAseq datasets generated in this study have been deposited in the GEO database under accession code GSE153615. Patient cohorts data analysed, including ovarian cancer (GSE26712)^48^, lymphoma (GSE10846)^49^, and breast cancer (GSE9893)^50^, are publicly available from the GEO database. Patient cohort data of PD-1/PD-L1 blockade therapy in melanoma is publicly available from NCBI BioProject database (PRJEB23709)^51^. The human macrophage transcriptome dataset is publicly available from the GEO database (GSE35449)^46^. TIDE computational method analyses were conducted on TIDE website (http://tide.dfci.harvard.edu)^45^. The remaining data associated with this study are presented in the Article or Supplementary Information. Further information and requests may be directed to and will be fulfilled by the corresponding author, Lili Yang (liliyang@ucla.edu). Source data are provided with this paper.

## Source data

Source Data
