# Supplementary material for: Targeting monoamine oxidase A-regulated tumor-associated macrophage polarization for cancer immunotherapy
Source: Nat Commun. 2021 Jun 10;12:3530. doi: 10.1038/s41467-021-23164-2 (PMC8192781; doi:10.1038/s41467-021-23164-2)
Supplement: Supplementary file 1 — Supplementary Information [file 41467_2021_23164_MOESM1_ESM.pdf]

Supplementary information for

Targeting monoamine oxidase A-regulated tumor-associated macrophage polarization for cancer immunotherapy

Authors: Yu-Chen Wang[1], Xi Wang[1], Jiaji Yu[1], Feiyang Ma[2], Zhe Li[1], Yang Zhou[1], Samuel Zeng[1], Xiaoya Ma[1], Yan-Ruide Li[1], Adam Neal[3,4], Jie Huang[1], Angela To[1], Nicole Clarke[1], Sanaz Memarzadeh[3,4,5,6,7], Matteo Pellegrini[2] & Lili Yang[1,3,6,7,*].

Affiliations:

[1]Department of Microbiology, Immunology and Molecular Genetics. University of California, Los Angeles, CA, USA.

[2]Department of Molecular, Cell and Developmental Biology, and Institute for Genomics and Proteomics, University of California, Los Angeles, CA, USA.

[3]Eli and Edythe Broad Center of Regeneration Medicine and Stem Cell Research. University of California, Los Angeles, CA, United States.

[4]Department of Obstetrics and Gynecology, David Geffen School of Medicine, University of California, Los Angeles, Los Angeles, CA, USA

[5]The VA Greater Los Angeles Healthcare System, Los Angeles, CA, USA

[6]Jonsson Comprehensive Cancer Center, the David Geffen School of Medicine, University of California, Los Angeles, CA, USA.

[7]Molecular Biology Institute, University of California, Los Angeles, CA, USA.

*Corresponding author. E-mail: liliyang@ucla.edu

The SI includes Supplementary figures 1-8 and Supplementary tables 1-3.

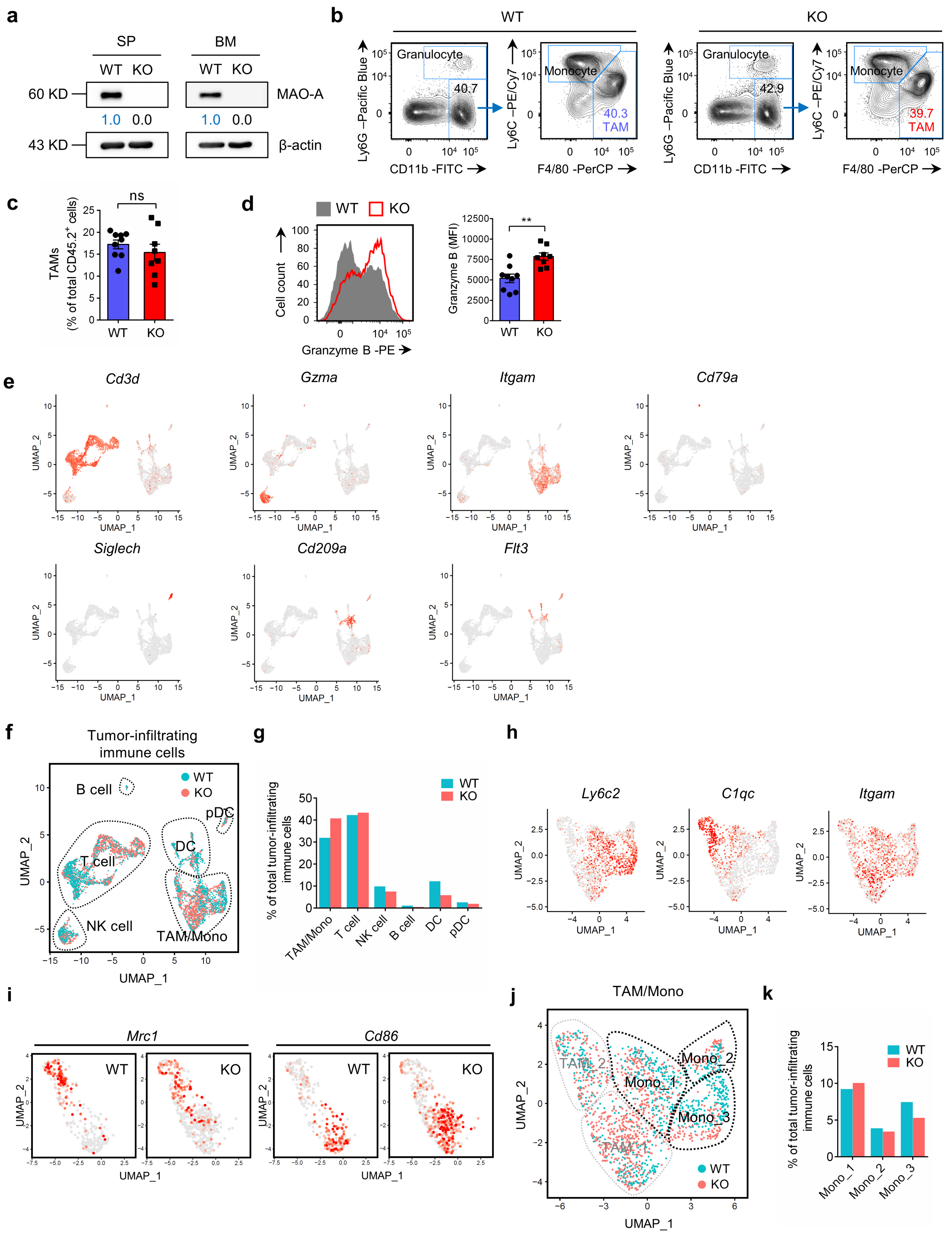

Supplementary Figure 1. MAO-A-deficient mice show reduced tumor growth associated with altered TAM polarization.

a, Western blot analyses of MAO-A protein expression in spleen (SP) and bone marrow (BM) cells harvested from *Maoa* WT (WT) and *Maoa* KO (KO) mice. Source data are provided as a Source Data file.

b-d, Phenotypes of TAMs and tumor-infiltrating $CD8^+$ T cells isolated from *Maoa* WT and *Maoa* KO mice bearing B16-OVA tumors, at day 18 post tumor challenge (WT, n = 9; KO, n = 8). (**b**) FACS gating strategy to identify TAMs (gated as $CD45.2^+CD11b^+Ly6G^-Ly6C^{-/low}F4/80^+$ cells) from total tumor-infiltrating immune cells (TIIs). (**c**) FACS quantification of TAMs. (**d**) FACS analyses of intracellular Granzyme B production in tumor-infiltrating $CD8^+$ T cells (gated as $CD45.2^+TCR\beta^+CD8^+$ cells from total TIIs) (**p = 0.0015).

e-k, scRNAseq analyses of TIIs isolated from *Maoa* WT or *Maoa* KO mice bearing B16-OVA tumors, at day 14 post tumor challenge. Uniform Manifold Approximation and Projection (UMAP) plots are presented. Each dot represents one single cell and is colored according to the expression level of an indicated gene. (**e**) UMAP of single TIIs, showing the expression patterns of 7 marker genes (*Cd3d*, *Gzma*, *Itgam*, *Cd79a*, *Siglech*, *Cd209a*, and *Flt3*) used to define 6 cell clusters (TAM/Mono, T cell, NK cell, B cell, DC, and pDC). TAM, tumor-associated macrophage; Mono, monocyte; NK, natural killer cell; DC, dendritic cell; pDC, plasmacytoid dendritic cell. **(f)** UMAP of single TIIs showing the formation of 6 cell clusters (TAM/Mono, T cell, NK cell, B cell, DC, and pDC). Each dot represents one single TII and is colored according to *Maoa* WT (blue) and *Maoa* KO (red) mice originalities. **(g)** Quantification of (f). (**h**) UMAP of single cells of the TAM/Mono subpopulation, showing the expression patterns of 3 marker genes (*Ly6c2*, *C1qc*, and *Itgam*) used to define 5 cell clusters (TAM_1, TAM_2, Mono_1, Mono_2, and Mono_3). (**i**) UMAP of single cells of the TAM_1 and TAM_2 subpopulations, showing the expression patterns of a pair of immunosuppressive and immunostimulatory signature genes (*Mrc1* and *Cd86*, respectively). (**j**) UMAP of monocyte subpopulation showing the formation of 3 monocyte clusters (Mono_1, Mono_2, and Mono_3). Each dot represents one single cell and is colored according to *Maoa* WT (blue) and *Maoa* KO (red) mice originalities. (**k**) Quantification of (j).

Representative of 1 (**e-k**), 2 (**a**), and 5 (**b-d**) experiments. All data are presented as the mean ± SEM. ns, not significant, $**p < 0.01$, by Student's *t* test. Statistics are all two-sided. Source data are provided as a Source Data file.

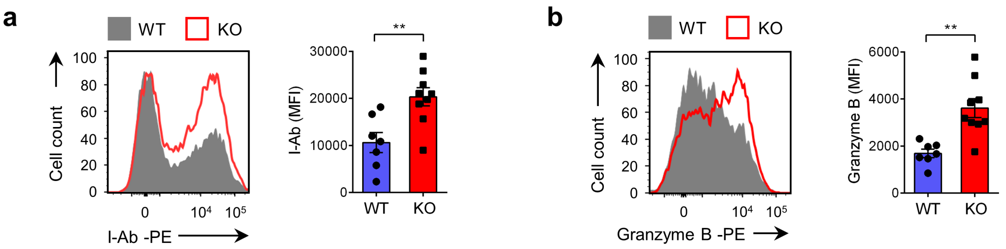

Supplementary Figure 2. MAO-A directly regulates TAM polarization and influences TAM-associated antitumor T cell reactivity.

BoyJ (CD45.1) wildtype (WT) mice reconstituted with bone marrow cells harvested from *Maoa* WT or *Maoa* KO donor mice (denoted as WT or KO mice, respectively) were inoculated with B16-OVA tumor cells. At day 18 post tumor challenge, TIIs were isolated from the experimental mice for FACS analysis.

a, FACS analyses of I-Ab expression on TAMs (gated as $CD45.2^+CD11b^+Ly6G^{-/low}F4/80^+$ cells of total TIIs) (**p = 0.0046). WT, n = 7; KO, n = 9.

b, FACS analyses of Granzyme B intracellular production in tumor-infiltrating $CD8^+$ T cells (gated as $CD45.2^+TCR\beta^+CD8^+$ cells of total TIIs) (**p = 0.0014). WT, n = 7; KO, n = 9.

Representative of 3 experiments. All data are presented as the mean ± SEM. **$p < 0.01$, by Student's *t* test. Statistics are all two-sided. Source data are provided as a Source Data file.

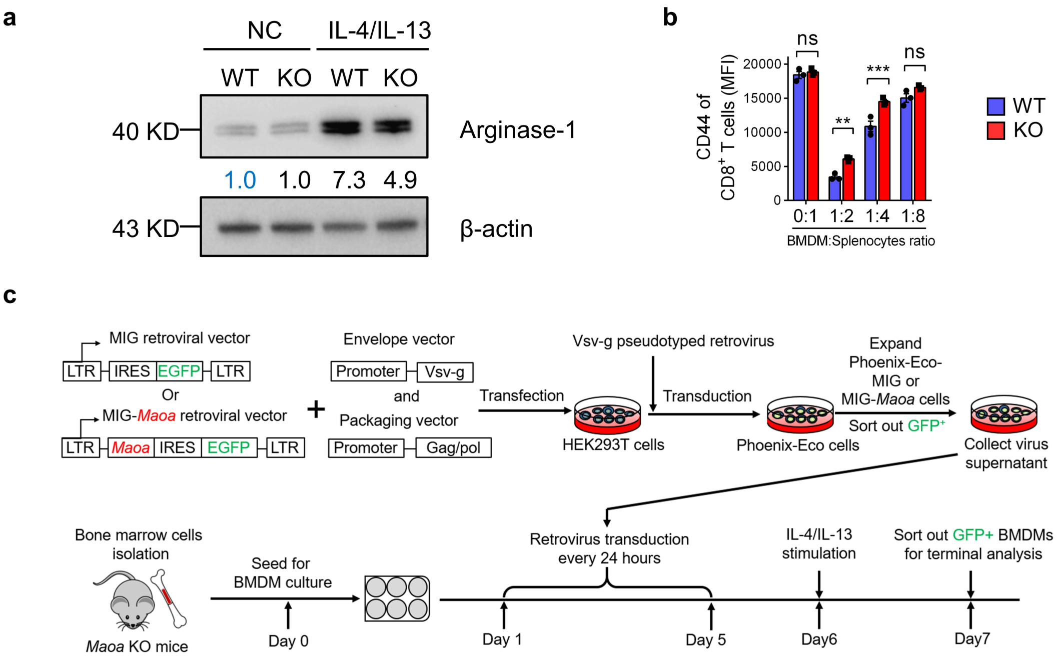

Supplementary Figure 3. MAO-A promotes macrophage immunosuppressive polarization.

a, Western blot analyses of Arginase-1 protein expression in *Maoa* WT and *Maoa* KO BMDMs, with or without IL-4/IL-13 polarization. BMDMs were treated with IL-4/IL-13 for 24 hours. BMDMs, bone marrow-derived macrophages; NC, no cytokine control BMDMs; IL-4/IL-13, IL-4 and IL-13 polarized BMDMs. Source data are provided as a Source Data file.

b, Studying the T cell suppression function of *Maoa* WT (WT) and *Maoa* KO (KO) IL-4/IL-13-polarized BMDMs in an *in vitro* macrophage/T cell co-culture assay (n = 3). Polarized BMDMs were mixed with 1 x 10^6 splenocytes harvested from B6 WT mice at 0:1, 1:2, 1:4, or 1:8 ratios. FACS quantifications of CD44 expression on $CD8^+$ T cells (identified as $CD11b^-TCR\beta^+CD8^+$ cells) are presented (1:2, **$p = 0.0028$; 1:4, ***$p < 0.001$).

c, Schematics showing the experimental design to overexpress MAO-A in *Maoa* KO BMDMs.

Representative of 2 (**a**) and 3 (**b**) experiments. All data are presented as the mean ± SEM. ns, not significant, **$p < 0.01$, ***$p < 0.001$, by 2-way ANOVA (A). Source data are provided as a Source Data file.

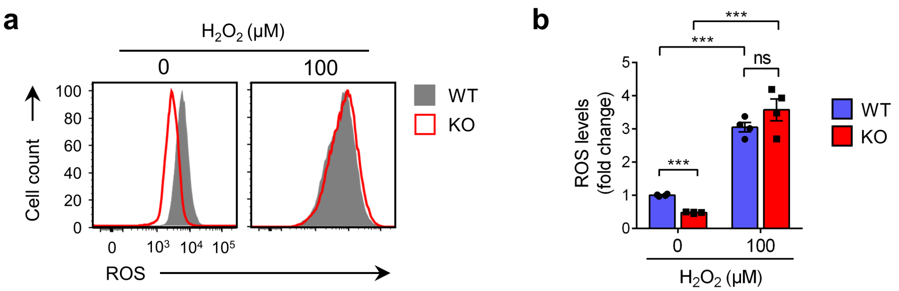

Supplementary Figure 4. MAO-A promotes macrophage immunosuppressive polarization via ROS upregulation.

Maoa WT and *Maoa* KO BMDMs (denoted as WT and KO, respectively) were treated with H_2O_2 for 30 minutes followed by IL-4/IL-13 stimulation for another 30 minutes. BMDMs were then collected for FACS analysis. N = 4.

a, FACS plots showing ROS levels in the indicated BMDMs.

b, Quantification of A. ***$p < 0.001$.

Representative of 2 experiments. All data are presented as the mean ± SEM. ns, not significant, ***$p < 0.001$, by 2-way ANOVA (**b**). Source data are provided as a Source Data file.

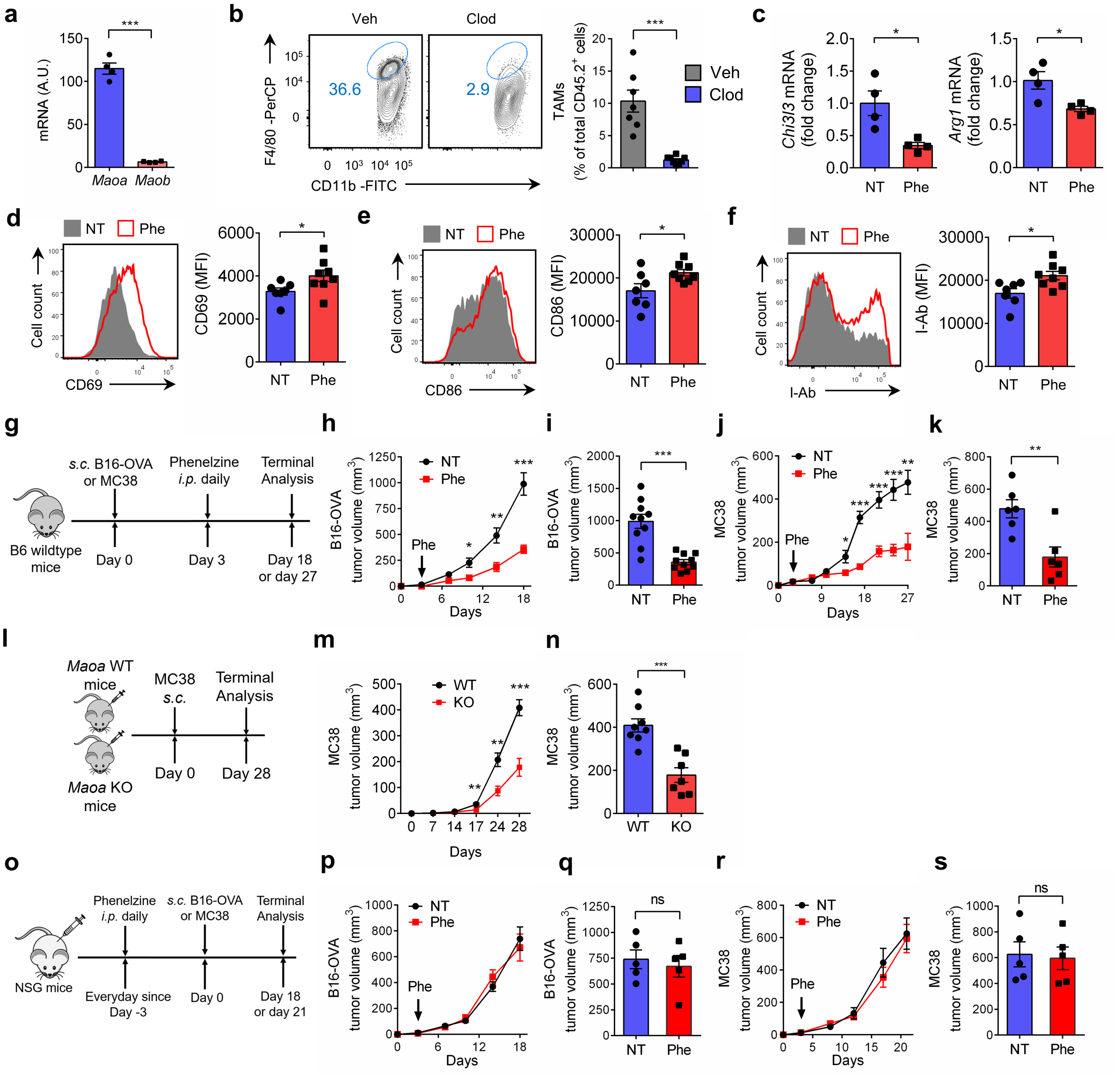

Supplementary Figure 5. MAO-A blockade for cancer immunotherapy- syngeneic mouse tumor model studies.

a, QPCR analyses of *Maoa* and *Maob* gene mRNA expression in B6 WT mice BMDMs (n = 4) (***$p <$ 0.001).

b, Efficient depletion of TAMs in B6 WT mice bearing B16-OVA tumors through clodronate liposome treatment (Clod). Tumor-bearing mice treated with vehicle liposomes (Veh) were included as a control. The experimental design is shown in main Figure 5F. FACS quantifications of TAMs (gated as

$CD45.2^+CD11b^+Ly6G^-Ly6C^{-/low}F4/80^+$ cells of total TIIs) are presented (Veh, n = 7; Clod, n = 8) (***$p < 0.001$).

c, QPCR analyses of *Chi3l3* and *Arg1* mRNA expression in TAMs isolated from B6 WT mice bearing B16-OVA tumors with or without phenelzine treatment (Phe or NT), TAMs were FACS purified from experimental mice at day 18 after tumor challenge (*Chi3l3,* *$p = 0.016$; *Arg1,* *$p = 0.0198$). N = 4.

d-f, FACS analyses of CD69 (**d**), CD86 (**e**), and I-Ab (**f**) expression on TAMs from B6 WT mice bearing B16-OVA tumors with or without phenelzine treatment (Phe or NT), TIIs were isolated from experimental mice at day 18 after tumor challenge (CD69, *$p = 0.0435$; CD86, *$p = 0.0303$; I-Ab, *$p = 0.0148$). TAMs were gated as $CD45.2^+CD11b^+Ly6G^-Ly6C^{-/low}F4/80^+$ cells. MFI, mean fluorescence intensity. NT, n = 7; Phe, n = 8.

g-k, Studying B16-OVA and MC38 tumor growth in B6 WT mice with or without phenelzine treatment (Phe or NT). (**g**) Experimental design. (**h**) B16-OVA tumor growth over time (n = 10). (**i**) B16-OVA tumor volume at day 18 (***$p < 0.001$). (**j**) MC38 tumor growth over time (n = 6). (**k**) MC38 tumor volume at day 27 (**$p = 0.0051$).

l-n, Studying MC38 tumor growth in *Maoa* WT and KO mice. (**l**) Experimental design. (**m**) MC38 tumor growth over time. (**n**) MC38 tumor volume at day 28 (***$p < 0.001$). WT, n = 8; KO, n = 7.

o-s, Studying B16-OVA and MC38 tumor growth in NSG mice with or without phenelzine treatment (Phe or NT; n = 5). (**o**) Experimental design. (**p**) B16-OVA tumor growth. (**q**) B16-OVA tumor volume at day 18. (**r**) MC38 tumor growth. (**s**) MC38 tumor volume at day 21.

Representative of 2 (**a-k**) and 4 (**l-s**) experiments. All data are presented as the mean ± SEM. ns, not significant, *$p < 0.05$, **$p < 0.01$, ***$p < 0.001$, by Student's *t* test (**a, b, c, d, e, f, i, k, n, q, s**). Statistics were all two-sided. Source data are provided as a Source Data file.

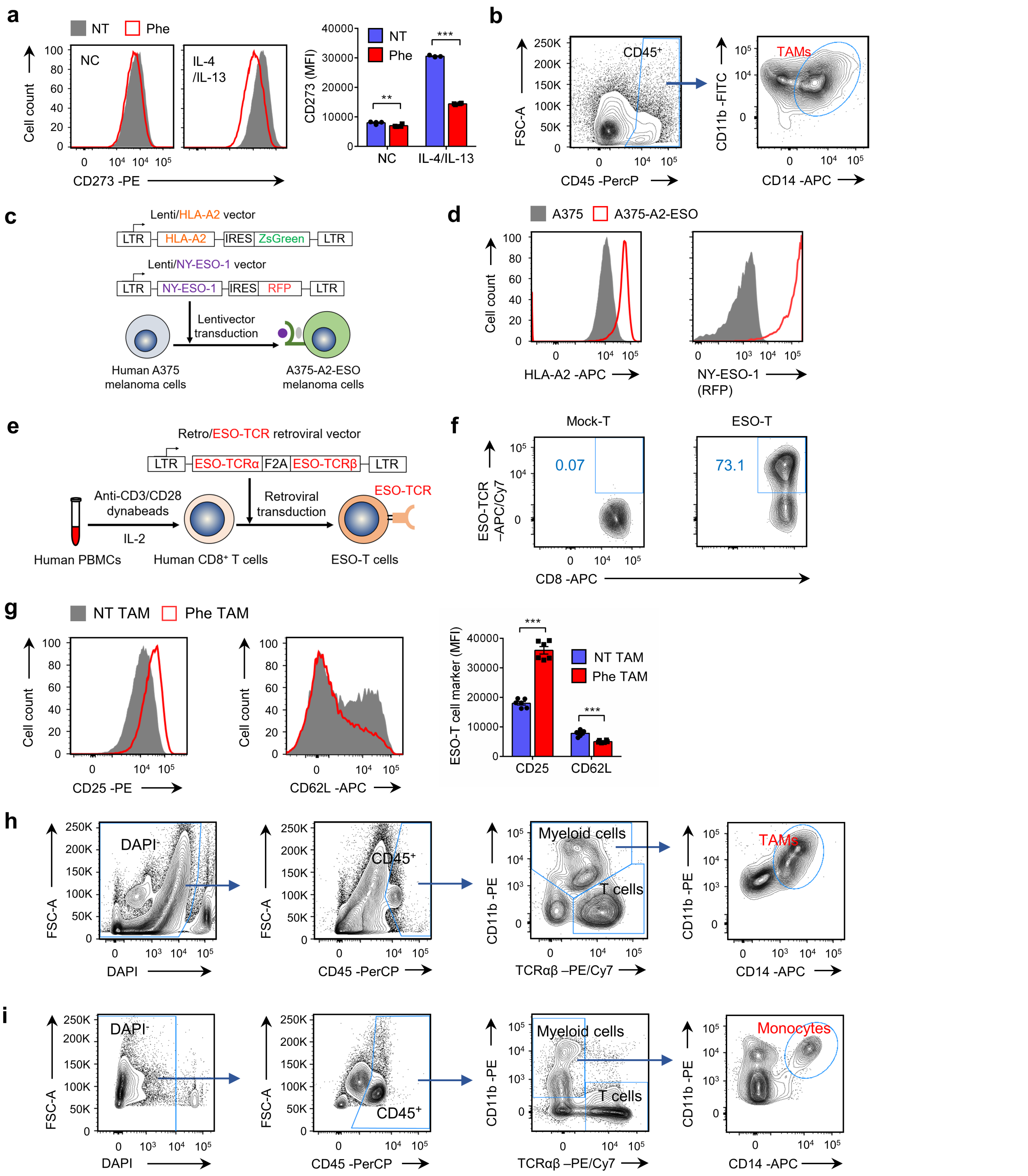

Supplementary Figure 6. MAO-A blockade for cancer immunotherapy- human TAM and clinical data correlation studies.

a, Studying the IL-4/IL-13-induced *in vitro* polarization of human monocyte-derived macrophages (MDMs) in the presence or absence of phenelzine (Phe) treatment. NC, no cytokine treatment; NT, no

Phe treatment. FACS analyses of CD273 expression on MDMs are presented (n = 3) (NC, ***p* = 0.0033; IL-4/IL-13, ****p* < 0.001).

b, FACS gating strategy to identify human TAMs (gated as $hCD45^+hCD11b^+hCD14^+$ cells of total TIIs) in a human Tumor-TAM Co-Inoculation xenograft mouse model. The experimental design is shown in main Figure 6h.

c,d, Generation of the A375-A2-ESO human melanoma cell line. (**c**) Experimental design. The A375-A2-ESO cell line was generated by stably co-transducing the parental A375 human melanoma cell line with a Lenti/HLA-A2 lentivector encoding the human HLA-A2 molecule and a Lenti/NY-ESO-1 lentivector encoding the human NY-ESO-1 tumor antigen. (**d**) FACS plots showing the detection of HLA-A2 molecule and NY-ESO-1 tumor antigen (indicated by RFP) on A375-A2-ESO cells. The parental A375 cells were included as a staining control.

e,f, Generation of the ESO-T cells. (**e**) Experimental design. Human peripheral blood mononuclear cells (hPBMCs) from healthy donors were stimulated *in vitro* with anti-CD3/CD28 and IL-2 to expand human $CD8^+$ T cells, followed by transduction with a Retro/ESO-TCR retrovector encoding an HLA-A2-restricted NY-ESO-1 specific TCR (clone 3A1). The resulting human $CD8^+$ T cells, denoted as the ESO-T cells, can specifically target the A375-A2-ESO human melanoma cells. (**f**) FACS plots showing the transduction efficiency of the engineered human $CD8^+$ ESO-T cells. Human $CD8^+$ T cells that received mock transduction were included as a staining control (denoted as Mock-T).

g, Studying the *in vitro* efficacy of phenelzine in reprogramming human TAMs and enhancing human T cell antitumor reactivity in an *in vitro* 3D human tumor/TAM/T cell organoid culture. The experimental design is shown in main Figure 6k. FACS plots showing the surface expression of CD25 and CD62L on ESO-T cells (n = 6). ****p* < 0.001.

h, FACS sorting of human TAMs from primary ovarian cancer patient tumor samples. Tumor-infiltrating immune cells were isolated from fresh ovarian cancer patient tumor samples and then were subjected to

FACS sorting to isolate TAMs (identified as DAPI$^-$hCD45$^+$hCD11b$^+$hTCRαβ$^-$hCD14$^+$ cells). Representative FACS plots are presented (n = 4).

i, FACS sorting of primary human monocytes from random healthy donor blood samples. PBMCs were subjected to FACS sorting to isolate monocytes (identified as DAPI$^-$hCD45$^+$hCD11b$^+$hTCRαβ$^-$hCD14$^+$ cells). Representative FACS plots are presented (n = 10).

Representative of 3 (**a-g**) and 4 (**h-i**) experiments. All data are presented as the mean ± SEM. $^{**}p < 0.01$, $^{***}p < 0.001$, by 2-way ANOVA (**a, g**). Source data are provided as a Source Data file.

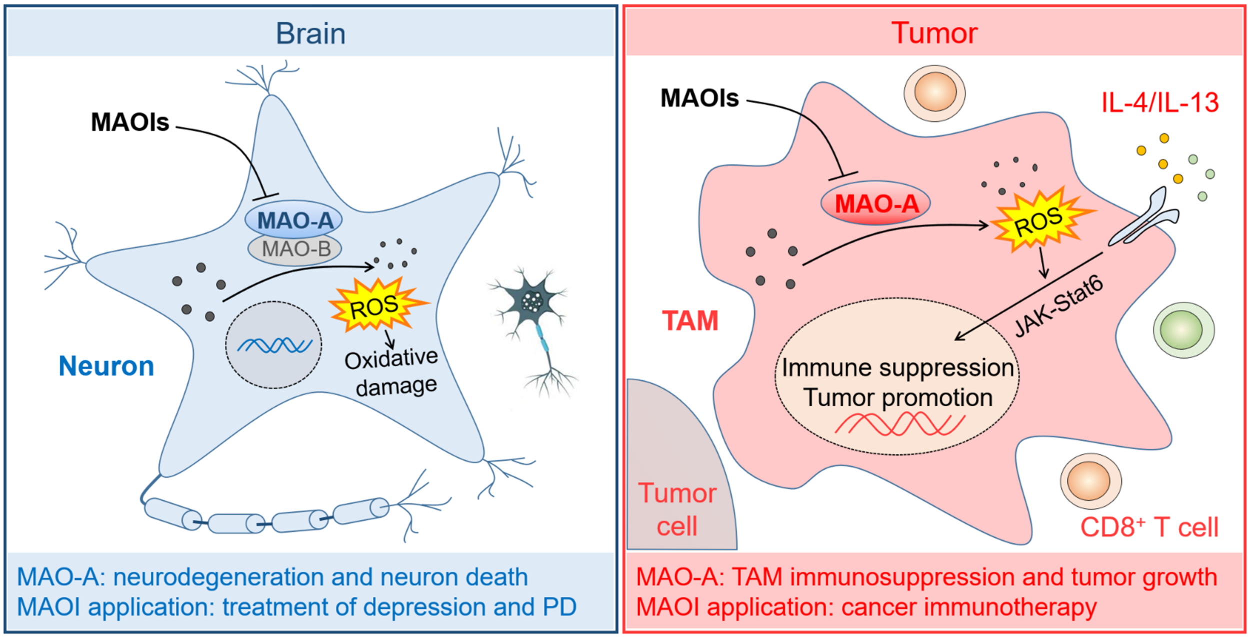

Supplementary Figure 7. The "intratumoral MAO-A-ROS axis" model.

Schematics showing the "intratumoral MAO-A-ROS axis" model. (Left Panel) Function of MAO-A in the brain. Neurons express MAO-A (as well as its isoenzyme MAO-B) that degrades monoamine neurotransmitters (e.g., dopamine, noradrenaline, and serotonin), thereby regulating neuron signal transmission. Meanwhile, the enzymatic activity of MAO-A generates hydrogen peroxide as a byproduct and thereby upregulating ROS levels (hence, oxidative stress) in neurons. Excessive oxidative stress induces the destruction of neuron cellular components and ultimately leading to neurodegeneration and neuron death. Small molecule monoamine oxidase inhibitors (MAOIs) have been developed and clinically utilized for treating neuropsychiatric disorders, such as depression, and neurodegeneration diseases, such as Parkinson's disease. (Right Panel) Function of MAO-A in a tumor. Analogous to neurons in the brain, TAMs in the tumor microenvironment also express MAO-A, that controls TAM intracellular ROS levels by hydrogen peroxide production, thereby regulating TAM immunosuppressive polarization and subsequently $CD8^+$ T cell antitumor reactivity. Established MAOI antidepressants can potentially be repurposed for improving cancer immunotherapy, through targeting the "MAO-A-ROS axis" of TAM polarization in tumors. Notably, unlike neurons that co-express MAO-A and MAO-B, TAMs in particular the immunosuppressive TAMs predominantly express MAO-A.

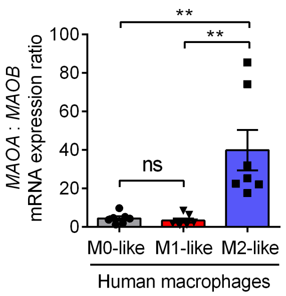

Supplementary Figure 8. *MAOA*:*MAOB* gene expression profile in human macrophages.

Comparing *MAOA*:*MAOB* gene expression ratio in human M0-, M1- and M2-like macrophages by analyzing a transcriptome data set (GSE35449). Each dot represents one single sample (n = 7) (M0-like:M2-like, $**p = 0.0019$; M1-like:M2-like, $**p = 0.0014$).

All data are presented as the mean ± SEM. ns, not significant, by 1-way ANOVA. Source data are provided as a Source Data file.

Supplementary Table 1. Information for the ovarian cancer tumor samples.

Sample number	Diagnosis	Tumor provided
1	Stage IIIC ovarian cancer (high grade serous adenocarcinoma type), status post neoadjuvant chemotherapy (3 cycles Carboplatin/Taxol)	Ovarian tumor
2	Synchronous stage IA ovarian adenocarcinoma and stage IA uterine adenocarcinoma (endometrioid type, FIGO grade 1)	Adnexal tumor
3	Dedifferentiated carcinoma, FIGO grade III	Ovarian tumor
4	High grade serous ovarian carcinoma, stage IIIC	Omental tumor

Supplementary Table 2. Reagent information.

REAGENT	SOURCE	IDENTIFIER	DILUTION
Antibodies			
Purified Anti-Mouse CD16/CD32 (Mouse Fc Block)	BD Biosciences	CAT#553142; RRID: AB_394657	1:50
Anti-mouse CD4 Antibody (Clone GK1.5)	Biolegend	CAT#100428; RRID: AB_493647	1:500
Anti-mouse CD8a Antibody (Clone 53-6.7)	Biolegend	CAT#100732; RRID: AB_893423	1:500
Anti-mouse CD25 Antibody (Clone PC61)	Biolegend	CAT#102008; RRID: AB_312857	1:100
Anti-mouse TCR β chain Antibody (Clone H57-597)	Biolegend	CAT#109220; RRID: AB_893624	1:200
Anti-mouse/human CD44 Antibody (Clone IM7)	Biolegend	CAT#103006; RRID: AB_312957	1:2000
Anti-mouse CD62L Antibody (Clone MEL-14)	Biolegend	CAT#104412; RRID: AB_313099	1:2000
Anti-mouse CD45.2 Antibody (Clone 104)	Biolegend	CAT#109830; RRID: AB_1186098	1:500
Anti-mouse CD45.1 Antibody (Clone A20)	Biolegend	CAT#110716; RRID: AB_313505	1:500
Anti-human/mouse Granzyme B Recombinant Antibody (Clone QA16A02)	Biolegend	CAT#372208; RRID: AB_2687032	1:100
Anti-mouse F4/80 Antibody (Clone BM8)	Biolegend	CAT#123126; RRID: AB_893483	1:200
Anti-mouse/human CD11b Antibody (Clone M1/70)	Biolegend	CAT#101206; RRID: AB_312789	1:1000
Anti-mouse Ly-6C Antibody (Clone HK1.4)	Biolegend	CAT#128018; RRID: AB_1732082	1:2000
Anti-mouse CD69 Antibody (Clone H1.2F3)	Biolegend	CAT#104508; RRID: AB_313111	1:200
Anti-mouse I-Ab Antibody (Clone AF6-120.1)	Biolegend	CAT#116420; RRID: AB_10575296	1:2000
Anti-mouse CD86 Antibody (Clone GL-1)	Biolegend	CAT#105012; RRID: AB_493342	1:1000
Anti-mouse Ly-6G Antibody (Clone 1A8)	Biolegend	CAT#127612; RRID: AB_2251161	1:500
Anti-mouse CD206 (MMR) Antibody (Clone C068C2)	Biolegend	CAT#141720; RRID: AB_2562248	1:200
Human Fc Receptor Blocking Solution (TrueStain FcX)	Biolegend	CAT#422302	1:50
Anti-human TCR(alpha)(beta) (Clone I26)	Biolegend	CAT#306716, RRID: AB_1953257	1:50
Anti-human CD45 (Clone H130)	Biolegend	CAT#304026, RFID: AB_893337	1:100
Anti-human CD4 (Clone OKT4)	Biolegend	CAT#317414, RRID: AB_571959	1:400

Anti-human CD8 (Clone SK1)	Biolegend	CAT#344714, RRID: AB_2044006	1:200
Anti-human CD14 (Clone HCD14)	Biolegend	CAT#325608, RRID: AB_830681	1:200
Anti-human CD11b (Clone ICRF44)	Biolegend	CAT#301330, RRID: AB_2561703	1:100
Anti-human CD206 (MMR) Antibody (Clone 15-2)	Biolegend	CAT#321110, RRID: AB_571885	1:100
Anti-human CD62L Antibody (Clone DREG-56)	Biolegend	CAT#304810, RRID: AB_314470	1:100
Anti-human CD273 (B7-DC, PD-L2) Antibody (Clone 24F.10C12)	Biolegend	CAT#329606, RRID: AB_1089019	1:100
Rat IgG2b, κ isotype control antibody (Clone eB149/10H5)	eBioscience	CAT#17-4031-82, RRID: AB_470176	1:100
Mouse IgG2b, κ isotype control antibody (Clone MPC-11)	Biolegend	CAT#400320	1:100
Jak1 (6G4) Rabbit mAb	Cell Signaling	CAT#3344	1:2000
Phospho-Jak1(Tyr1034/1035) (D7N4Z) Rabbit mAb	Cell Signaling	CAT#74129	1:2000
Jak2 (D2E12) XP® Rabbit mAb	Cell Signaling	CAT#3230	1:2000
Phospho-Jak2 (Tyr1008) (D4A8) Rabbit mAb	Cell Signaling	CAT#8082	1:2000
Jak3 (D7B12) Rabbit mAb	Cell Signaling	CAT#8863	1:2000
Phospho-Jak3 (Tyr980/981) (D44E3) Rabbit mAb	Cell Signaling	CAT#5031	1:2000
Phospho-Stat6 (Tyr641) (D8S9Y) Rabbit mAb	Cell Signaling	CAT#56554	1:2000
Stat6 (D3H4) Rabbit mAb	Cell Signaling	CAT#5397	1:2000
Arginase-1 (D4E3M™) XP® Rabbit mAb	Cell Signaling	CAT#93668	1:2000
Anti-rabbit IgG, HRP-linked Antibody	Cell Signaling	CAT#7074	1:5000
Rabbit Anti-Monoamine Oxidase A/MAO-A antibody (EPR7101)	Abcam	CAT#ab126751	1:2000
β-Actin Antibody (AC-15)	Santa Cruz	CAT#sc-69879	1:5000
Anti-mouse IgG, HRP-linked Antibody	Cell Signaling	CAT#7076	1:5000

Supplementary Table 3. Primers used for QPCR.

Gene	Forward Primer 5'-3'	Reverse Primer 5'-3'
Ube2d2	ACAAGGAATTGAATGACCTGGC	CACCCTGATAGGGGCTGTC
Mrc1	CTCTGTTCAGCTATTGGACGC	CGGAATTTCTGGGATTCAGCTTC
Chi3l3	CAGGTCTGGCAATTCTTCTGAA	GTCTTGCTCATGTGTGTAAGTGA
Arg1	TGTCCCTAATGACAGCTCCTT	GCATCCACCCAAATGACACAT
Il6	CTGCAAGAGACTTCCATCCAG	AGTGGTATAGACAGGTCTGTTGG
Tnf	AAGCCTGTAGCCCACGTCGTA	AGGTACAACCCATCGGCTGG
Ccl2	TTAAAAACCTGGATCGGAACCAA	GCATTAGCTTCAGATTTACGGGT
Maoa	CCTGGTATCATGACTCTGTATGG	CTTGGACTCAGGCTCTTGAAC
Maob	ATGAGCAACAAAAGCGATGTGA	TCCTAATTGTGTAAGTCCTGCCT
Maoa (for MIG-*Maoa* retrovector)	GTACCCGCTTTGGAGATAAC	GAAGGGCCACTGATGTGGAA
ACTB	GAGCACAGAGCCTCGCCTTT	ACATGCCGGAGCCGTTGTC
MAOA	GTCTTAAATGGTCTCGGGAAGG	CCAGAAGGTGTGGGTGATTT
ALOX15	GGGCAAGGAGACAGAACTCAA	CAGCGGTAACAAGGGAACCT
CD200R1	TGGTTGTTGAAAGTCAATGGCT	CTCAGATGCCTTCACCTTGTTT
