# Supplementary material for: Targeting monoamine oxidase A-regulated tumor-associated macrophage polarization for cancer immunotherapy
Source: Nat Commun. 2021 Jun 10;12:3530. doi: 10.1038/s41467-021-23164-2 (PMC8192781; doi:10.1038/s41467-021-23164-2)
Supplement: Supplementary file 2 — Reporting Summary [file 41467_2021_23164_MOESM2_ESM.pdf]

nature research

Corresponding author(s): Lili Yang

Last updated by author(s): Feb 9, 2021

Reporting Summary

Nature Research wishes to improve the reproducibility of the work that we publish. This form provides structure for consistency and transparency in reporting. For further information on Nature Research policies, see our Editorial Policies and the Editorial Policy Checklist.

Statistics

For all statistical analyses, confirm that the following items are present in the figure legend, table legend, main text, or Methods section.

n/a	Confirmed	
☐	☒	The exact sample size (n) for each experimental group/condition, given as a discrete number and unit of measurement
☐	☒	A statement on whether measurements were taken from distinct samples or whether the same sample was measured repeatedly
☐	☒	The statistical test(s) used AND whether they are one- or two-sided<br>*Only common tests should be described solely by name; describe more complex techniques in the Methods section.*
☐	☒	A description of all covariates tested
☐	☒	A description of any assumptions or corrections, such as tests of normality and adjustment for multiple comparisons
☐	☒	A full description of the statistical parameters including central tendency (e.g. means) or other basic estimates (e.g. regression coefficient) AND variation (e.g. standard deviation) or associated estimates of uncertainty (e.g. confidence intervals)
☐	☒	For null hypothesis testing, the test statistic (e.g. F, t, r) with confidence intervals, effect sizes, degrees of freedom and P value noted<br>*Give P values as exact values whenever suitable.*
☒	☐	For Bayesian analysis, information on the choice of priors and Markov chain Monte Carlo settings
☒	☐	For hierarchical and complex designs, identification of the appropriate level for tests and full reporting of outcomes
☒	☐	Estimates of effect sizes (e.g. Cohen's d, Pearson's r), indicating how they were calculated

Our web collection on statistics for biologists contains articles on many of the points above.

Software and code

Policy information about availability of computer code

Data collection	Flow Cytometry: MACSQuant Analyzer 10 Flow Cytometer (Miltenyi Biotec);<br>FACSAria II Flow Cytometer (BD Biosciences);<br>Single cell RNA sequencing: Novaseq 6000 Sequencing System (Illumina);<br>qPCR:7500 Real-time PCR System (Applied Biosystems);<br>Westernblot: ChemiDoc Imaging System (Bio-rad)
Data analysis	FlowJo Software V10 (BD Biosciences);<br>Rstudio (Rstudio);<br>GraphPad Prism 6 (GraphPad);<br>ImageJ (NIH);<br>Microsoft Excel (Microsoft)

For manuscripts utilizing custom algorithms or software that are central to the research but not yet described in published literature, software must be made available to editors and reviewers. We strongly encourage code deposition in a community repository (e.g. GitHub). See the Nature Research guidelines for submitting code & software for further information.

Data

Policy information about availability of data

All manuscripts must include a data availability statement. This statement should provide the following information, where applicable:

- Accession codes, unique identifiers, or web links for publicly available datasets
- A list of figures that have associated raw data
- A description of any restrictions on data availability

All data associated with this study are presented in the article or Supplemental Information. The scRNAseq datasets generated in this study have been deposited in the GEO database under accession code GSE153615 (https://www.ncbi.nlm.nih.gov/geo/query/acc.cgi?acc=GSE153615). Patient cohorts data analyzed, including ovarian cancer (https://www.ncbi.nlm.nih.gov/geo/query/acc.cgi?acc=GSE26712) (GSE26712), lymphoma (https://www.ncbi.nlm.nih.gov/geo/query/acc.cgi?acc=GSE10846) (GSE10846), and breast cancer (https://www.ncbi.nlm.nih.gov/geo/query/acc.cgi?acc=GSE9893) (GSE9893), are publicly available from the GEO database. Patient cohort data of PD-1/PD-L1 blockade therapy in melanoma is publicly available from NCBI BioProject database (https://www.ncbi.nlm.nih.gov/bioproject/PRJEB23709) (PRJEB23709). Human macrophage transcriptome data set is publicly available from the GEO database (https://www.ncbi.nlm.nih.gov/geo/query/acc.cgi?acc=GSE35449) (GSE35449). TIDE computational method analyses were conducted on TIDE website (http://tide.dfci.harvard.edu). Further information and requests for new reagents generated in this study may be directed to and will be fulfilled by the Lead Contact, Lili Yang (liliyang@ucla.edu).

Field-specific reporting

Please select the one below that is the best fit for your research. If you are not sure, read the appropriate sections before making your selection.

- [x] Life sciences
- [ ] Behavioural & social sciences
- [ ] Ecological, evolutionary & environmental sciences

For a reference copy of the document with all sections, see nature.com/documents/nr-reporting-summary-flat.pdf

Life sciences study design

All studies must disclose on these points even when the disclosure is negative.

Sample size	For all studies samples size were defined on the basis of previous studies in the laboratory. For in vivo studies, between 4 to 10 mice per group were used dependent on the experimental design and were able to reach a significant level of 0.05. For in vitro studies, at least 3 mice/biological group were used as cell donors with technical replicates that encompassed at least 2 wells per mouse. For human studies, at least 4 donors or patient samples per group were used.
Data exclusions	No data were excluded for analysis.
Replication	All experiments were performed independently. Replicates of each individual experiment was stated in its figure legends. The experimental findings were reproduced with similar results. For single cell RNA sequencing, a mixture of 10 mice TII suspensions were combined for each group.
Randomization	All mice were randomized. Within one experiment, all mice were age- and sex-matched. For human studies, all donors or patients are randomized.
Blinding	Experiments were not performed in a blinded fashion. For mice in vivo studies, the investigators were aware of genotyping results. All data were analyzed by software with objective standard.

Reporting for specific materials, systems and methods

We require information from authors about some types of materials, experimental systems and methods used in many studies. Here, indicate whether each material, system or method listed is relevant to your study. If you are not sure if a list item applies to your research, read the appropriate section before selecting a response.

Materials & experimental systems

n/a	Involved in the study	
☐	☒	Antibodies
☐	☒	Eukaryotic cell lines
☒	☐	Palaeontology and archaeology
☐	☒	Animals and other organisms
☐	☒	Human research participants
☒	☐	Clinical data
☒	☐	Dual use research of concern

Methods

n/a	Involved in the study	
☒	☐	ChIP-seq
☐	☒	Flow cytometry
☒	☐	MRI-based neuroimaging

Antibodies

Antibodies used	Fluorochrome-conjugated monoclonal antibodies specific for mouse CD45.2 (clone 104), CD11b (Clone M1/70), Ly6G (Clone 1A8), F4/80 (Clone BM8), Ly6C (Clone HK1.4), CD206 (Clone C068C2), CD69 (clone H1.2F3), CD86 (Clone GL-1), I-Ab (Clone AF6-120.1), TCR(clone H57-597), CD45.1 (Clone A20), CD4 (Clone GK1.5), CD8 (clone 53-6.7), CD25 (clone PC61), CD44 (clone IM7), CD62L (clone MEL-14), and Granzyme B (Clone QA16A02) were purchased from BioLegend. Mouse Fc Block (anti-mouse CD16/32; clone 2.4G2) was purchased from BD Biosciences.<br>Fluorochrome-conjugated monoclonal antibodies specific for human CD45 (clone H130), CD11b (Clone ICRF44), CD14 (Clone HCD14), CD206 (Clone 15-2), CD273 (Clone 24F.10C12), TCRalphabeta (clone I26), CD4 (clone OKT4), CD8 (clone SK1), CD44 (clone IM7), CD62L (clone DREG-56), and human Fc Receptor Blocking Solution (TruStain FcX™, 422302) were purchased from BioLegend.<br>Primary antibodies against mouse Stat6, p-Stat6 (Tyr641), JAK1, p-JAK1 (Tyr1034/1035), JAK2, p-JAK2 (Tyr1008), JAK3, p-JAK3 (Tyr980/981), Arginase-1, HRP-labeled anti-rabbit secondary antibodies, and HRP-labeled anti-mouse secondary antibodies were purchased from Cell Signaling Technology. MAO-A antibody was purchased from Abcam (Clone EPR7101). Primary antibodies against beta-actin (AC-15) were purchased from Santa Cruz Biotechnology.<br>For ICB therapy, aPD-1 blocking antibody (clone RMP1-14) and aPD-1 isotype control (rat IgG2a) were purchased from BioXCell.<br>Multiple lots were used for each antibody. A list of detailed information of antibodies used in this study (name, supplier, cat, clone ID) is provided in Supplementary Tables.
Validation	All antibodies were validated by the supplier. All respective validation data are available on the manufacturer's website.

Eukaryotic cell lines

Policy information about cell lines

Cell line source(s)	The B16-OVA mouse melanoma cell line and the PG13 retroviral packaging cell line were provided by Dr. Pin Wang (University of South California, CA). The MC-38 mouse colon adenocarcinoma cell line was provided by Dr. Antoni Ribas (UCLA). The HEK 293T and Phoenix-ECO retroviral packaging cell lines, the A375 human melanoma cell line, and the L-929 mouse connective tissue cell line were purchased from the American Type Culture Collection (ATCC). The A375-A2-ESO cell line was previously reported and was generated in the lab. The Phoenix-ECO-MIG, Phoenix-ECO-MIG-Maoa, and PG13-ESO-TCR stable virus producing cell lines were generated in this study. The MIG (MSCV-IRES-GFP) retroviral vector was previously reported and was generated in the lab. MIG-Maoa and Retro/ESO-TCR retroviral vectors were generated in this study.
Authentication	All cell lines were utilized within 10 passages of a master stock to ensure accuracy.
Mycoplasma contamination	All cell lines were tested negative for mycoplasma contamination.
Commonly misidentified lines (See ICLAC register)	No misidentified lines were used.

Animals and other organisms

Policy information about studies involving animals; ARRIVE guidelines recommended for reporting animal research

Laboratory animals	C57BL/6J (B6), B6.SJL-PtprcaPepcb/BoyJ (CD45.1, BoyJ), 129S-Maoatm1Shih/J (Maoa KO) 56, and NOD.Cg-Prkdcscid Il2rgtm1Wjl/SzJ (NSG) mice were purchased from the Jackson Laboratory (Bar Harbor). Maoa KO mice were backcrossed with C57BL/6J mice for more than 9 generations at the University of California, Los Angeles (UCLA). Eight- to twelve-week-old female mice were used for all experiments unless otherwise indicated.
Wild animals	*Provide details on animals observed in or captured in the field; report species, sex and age where possible. Describe how animals were caught and transported and what happened to captive animals after the study (if killed, explain why and describe method; if released, say where and when) OR state that the study did not involve wild animals.*
Field-collected samples	*For laboratory work with field-collected samples, describe all relevant parameters such as housing, maintenance, temperature, photoperiod and end-of-experiment protocol OR state that the study did not involve samples collected from the field.*
Ethics oversight	All animals were maintained at the UCLA animal facilities and all animal experiments were approved by the Institutional Animal Care and Use Committee of UCLA.

Note that full information on the approval of the study protocol must also be provided in the manuscript.

Human research participants

Policy information about studies involving human research participants

Population characteristics	Detailed patients information is provided in Supplementary Table, including collection date, age, diagnosis and staging.
Recruitment	Primary human ovarian cancer tumour samples were obtained from the operating room at the UCLA Medical Center from consenting patients using IRB-approved protocols (IRB# 10-000727).<br>Human peripheral blood mononuclear cells (PBMCs) of healthy donors were obtained from the CFAR Gene and Cellular Therapy Core Laboratory at UCLA, without identification information under federal and state regulations.

Ethics oversight	All human samples were obtained following institutional guidelines under protocols approved by the institutional review boards (IRBs) at the UCLA Medical Center.

Note that full information on the approval of the study protocol must also be provided in the manuscript.

Flow Cytometry

Plots

Confirm that:

- [x] The axis labels state the marker and fluorochrome used (e.g. CD4-FITC).
- [x] The axis scales are clearly visible. Include numbers along axes only for bottom left plot of group (a 'group' is an analysis of identical markers).
- [x] All plots are contour plots with outliers or pseudocolor plots.
- [x] A numerical value for number of cells or percentage (with statistics) is provided.

Methodology

Sample preparation	Solid tumours were collected from experimental mice at the termination of a tumour experiment. Tumours were cut into small pieces and smashed against a 70-m cell strainer (Corning, 07-201-431) to prepare single cells. Immune cells were enriched through gradient centrifugation with 45% Percoll (Sigma-Aldrich, P4937) at 800 g for 30 mins at 25 °C without braking, followed by treatment with Tris-buffered ammonium chloride buffer to lyse red blood cells according to a standard protocol (Cold Spring Harbor Protocols). The resulting TII isolates were then used for further analysis.
Instrument	MACSQuant® Analyzer 10 Flow Cytometer (Miltenyi Biotec); FACSAria II Flow Cytometer (BD Biosciences).
Software	FlowJo software V10 (BD Biosciences)
Cell population abundance	For cell sorting, highest purity method was used in FACSAria II Flow Cytometer and >98% post-sorting purity was verified through flow cytometry analysis.
Gating strategy	SSC-A/FSC-A, SSC-W/SSC-H and FSC-W/FSC-H were used to identify singlets. Fixable Viability Dye eFluor 506 (Biolegend) was used to exclude dead cells in flow analysis. DAPI (Thermo Fisher Scientific) was included to exclude dead cells in FACS sorting. Gating strategy for each cell subtype in each experiment was described in the article and provided as panels in supplementary figures.

- [x] Tick this box to confirm that a figure exemplifying the gating strategy is provided in the Supplementary Information.
